# Impact of non-pharmacological interventions on the first wave of COVID-19 in Portugal 2020

**DOI:** 10.1016/j.heliyon.2024.e41569

**Published:** 2025-01-31

**Authors:** Dinis B. Loyens, Constantino Caetano, Carlos Matias-Dias

**Affiliations:** a Unidade de Saúde Pública da Amadora, Unidade Local de Saúde Amadora/Sintra, Portugal; b Departamento de Epidemiologia, Instituto Nacional de Saúde Doutor Ricardo Jorge, Portugal

## Abstract

**Introduction:**

The COVID-19 pandemic caused over 7 million global deaths. Without vaccines during the first wave, governments implemented nonpharmacological interventions (NPIs) such as lockdowns, school closures, and travel restrictions. This study quantifies the impact of NPIs on COVID-19 transmission in Portugal between 24th February and 1st May.

**Methods:**

A compartmental SEIR (Susceptible, Exposed, Infectious, Removed) model was employed to simulate the first COVID-19 wave in Portugal, using a Bayesian approach and symptom-onset incidence data. The effect of the lockdown, which began on March 22, 2020, on the effective reproductive number, *R*_*t*_ was measured. A counterfactual scenario was created to ascertain the number of cases prevented by the NPIs during the first 15 days after the implementation of NPI.

**Results:**

The lockdown reduced overall transmission by 68·6 % (95%Credible Interval (95%CrI): 59·2 %; 77·5 %), almost immediately. This corresponds to a reduction in the effective reproductive number from 2·56 (95%CrI: 2·08; 3·40) to 0·80 (95%CrI: 0·76; 0·84). The counterfactual scenario estimated that the lockdown prevented 118052 (95%CrI: 99464; 145605) cases between 24th February and 6th April.

**Discussion:**

The lockdown significantly reduced COVID-19 transmission in Portugal, bringing Rt below 1, meaning each person infected fewer than one individual. While costly, lockdowns effectively control disease spread in the absence of vaccines.

**Conclusion:**

Our findings suggest NPIs curbed epidemic transmission, reducing Rt below 1 and easing hospital loads and deaths. This research will help inform future pandemic decision-making and infectious disease modeling worldwide.

## 1 Introduction

The first case of COVID-19 was reported in Wuhan (China) on December 31, 2019, having spread to around 200 countries and territories, causing more than 7 million deaths worldwide [1]. The WHO declared COVID-19 as a Public Health Emergency of International Concern on January 30, 2020 [2]. During the first COVID-19 outbreak, there was no vaccine available yet, therefore many governments implemented non-pharmacological interventions (NPIs) in order to curb disease spread and avoid overburdening healthcare services, such as lockdowns, closure of schools, restrictions on air travel, and mandatory remote work [[3], [4], [5]]. Nationwide lockdowns however come at a great cost to both the economy and the mental health of the population [6,7], so their effectiveness needs to be studied thoroughly to ensure they are implemented only when absolutely required. Other NPIs albeit not so extreme, also carry risks, for example, the delays in education and development of children due to the closing of schools and the requirement of parents to stay at home with their children [8,9]. There is ample literature that studies the effect of NPIs in Portugal using non-Bayesian statistical methods [[10], [11], [12]]. These methods were especially useful during the subsequent waves of the pandemics, allowing for an estimation of the overall effect of NPIs among the population, as they were easy to implement and understand, and there was an urgent need for knowledge about the impact of NPIs. After the pandemic, however, there is an opportunity to do a deeper study of these mechanisms, allowing a greater preparation in future pandemics. A Bayesian approach accounts for the uncertainty inherent to epidemiological data [13], making it particularly suitable for such analyses. Unlike more traditional methods, Bayesian inference models uncertainty, providing a probabilistic framework that combines prior knowledge with observed data [13]. This allows incorporating uncertainty in key parameters, such as disease transmission rates and the reproductive number, offering interval estimates rather than singlepoint values. By not relying entirely on single-point values, Bayesian methods provide more realistic inferences, particularly in the face of incomplete or noisy data, which is especially relevant to public health. In the context of this study, the Bayesian framework enables a deeper understanding of disease dynamics, improving the reliability of the conclusions drawn and contributing to the broader goal of optimizing public health responses in future outbreaks. Some studies have used publicly available data and Bayesian methods to estimate the impact of NPIs, such as Flaxman et al. [14], whose methods were the basis for this study, as well as others. However, the data used in this study is based on symptom onset, reducing the lag period in comparison with studies using data from hospitalizations or ICU cases [10]. This also minimizes variability introduced by batch reporting, especially during weekends, as the data was smoothed using statistical inference, previously to this study.

Worldwide pandemics and disease outbreaks, including communicable diseases for which no vaccine is available, are expected to increase due to climate change, migrations, and increasing air travel [15].

It is, therefore, important to study the impact of NPIs on disease spread to ensure that they are applied in a timely manner and maximize their effect. Many studies have been published demonstrating their positive impact on reducing transmission of COVID-19 in the absence of a vaccine [3,4]. This study aims to quantify the impact of lockdown on the first wave of COVID-19 in Portugal, using Bayesian methods and incidence data based on symptom onset on a SEIR (Susceptible, Exposed, Infectious, and Removed) model. This will result in more accurate and useful insights, which can influence policymaking and the design of future interventions.

## 2 Methods

$$\begin{array}{c} \frac{dS\left(t\right)}{dt}=-\beta \left(t\right)\frac{S\left(t\right)I\left(t\right)}{N}\\ \frac{dE\left(t\right)}{dt}=\beta \left(t\right)\frac{S\left(t\right)I\left(t\right)}{N}-\alpha E(t\\ \begin{array}{c} \frac{dI\left(t\right)}{dt}=\alpha E\left(t\right)-\gamma I(t\\ \frac{dR\left(t\right)}{dt}=\gamma I\left(t\right) \end{array} \end{array}$$

A compartmental SEIR model was used to simulate the first COVID-19 wave in Portugal in 2020. The following equations describe it:

Flaxman et al. [14] developed a function that describes the effect of an NPI on the transmission rate of a disease.

$$g\left(t\right)=\eta +\left(1-\eta \right)∙\frac{1}{1+e^{\xi \left(t-t_{1}-\nu \right)}}$$

This equation allows the estimation of the effective reproductive number, *R*_*t*_, (the average number of infections caused by an infected individual) and the final impact of the NPIs on *R*_*t*_ (*η*, as well as the time to reach 50 % of its effect *ν*. Table 1 describes the variables and parameters used in the model. The progression from exposed to infectious and recovery rates were obtained by calculating the inverse of the incubation and infectious periods, respectively.

**Table 1:** Variables and parameters used.

Variable	Description
S	Susceptible individuals
E	Exposed individuals
I	Infectious individuals
R	Removed individuals
β	Transmission rate
α	Progression from exposed to infectious rate
γ	Recovery rate
η	Final impact of the NPI
ν	The time between the start of the NPI and achieving 50 % of its total effect
t₁	NPI starts having effect
ξ	The slope of the transmission variation
μₜ	Daily absolute incidence

The lockdown started on March 22, 2020 [16], with immediate effect. Lockdown meant that people were required to stay at home at all times except for specific reasons such as: high-priority professions (healthcare, for example), medical care, walking the pets and buying groceries, therefore reducing contacts between individuals. The chosen day for the start of the NPIs effect (*t*_1_) was 22nd March and the measured effect encompasses not only the lockdown, which was the main reason for decreasing in contacts, but also the closing of schools, increase in remote work, and disease awareness. The data used for this study was the daily absolute incidence in Portugal between 22nd February and May 1, 2020, based on the date of symptom onset. This data was extracted from the Portuguese notifiable disease database (BI-SINAVE). Population data was obtained from the Portuguese National Statistics. The model was fitted to observed daily incidence data using the methodology proposed by Grinsztajn et al. [17]. Due to the scarcity of COVID-19 tests available and testing policy [18], there may have been an under-ascertainment of real cases among the population. According to the relevant literature, the reporting probability of a case, or the ascertainment rate, was between 22·0 % and 36·6 % [19]. The initial beliefs about each parameter (prior) were drawn from available data where possible and are described in Table 2. All parameters are positive and truncated at 0. The prior distribution chosen for the initial number of infectious and exposed individuals was based on observed data. The half reduction parameter (*ν*) was expected to occur until two days after the start of the intervention and the final impact of the NPI (*η*) was expected to be at least 50 %, based on previous literature [3,4]. More information about prior assumptions is available on the supplementary material.

**Table 2:** Priors used in the study.

Prior	Distribution	Reference
β	Normal(mean=2, SD=1)	Grinzstan et al. [1]
γ	Gamma(mean=4, var=20)	Byrne et al. [6]
α	Normal(mean=0.3, SD=0.001)	Byrne et al. [6]
1/φ	Exponential(rate=5)	Assumed
The initial number of infectious (i₀)	Normal(mean=1, SD=10)/Normal(mean=1, SD=2)	Assumed
The initial number of exposed (e₀)	Normal(mean=1, SD=10)/Normal(mean=1, SD=2)	Assumed
η	Beta(α=4, β=8)	Assumed
ν	Exponential(rate=1/2)	Assumed
ξ	Beta(α=1, β=1)	Assumed

A sensitivity analysis on the importance of the reporting probability was performed to ensure which values better fit the observed data. This sensitivity analysis is available in the supplementary materials. The projected daily incidence without any NPI was calculated by removing the effect of the NPI (such that *η* = 1 in a counterfactual scenario, thus allowing the calculation of prevented cases by the NPI during the first 15 days after 22nd of March. This counterfactual curve represents the daily incidence if the individual contact patterns stayed at pre-pandemic levels.

The posterior distributions were estimated using Markov Chain Monte Carlo (MCMC) algorithms, particularly Hamiltonian Monte Carlo (HMC). The model was calibrated using R, specifically R version 4.3.3 and the package rstan version 2.32.5. The model was written in STAN. A 95 % Credible Interval (95%CrI) was obtained for each quantity of interest.

The funders of the study had no role in study design, data collection, data analysis, data interpretation, or writing of the report.

## 3 Results

**Fig. 1:**
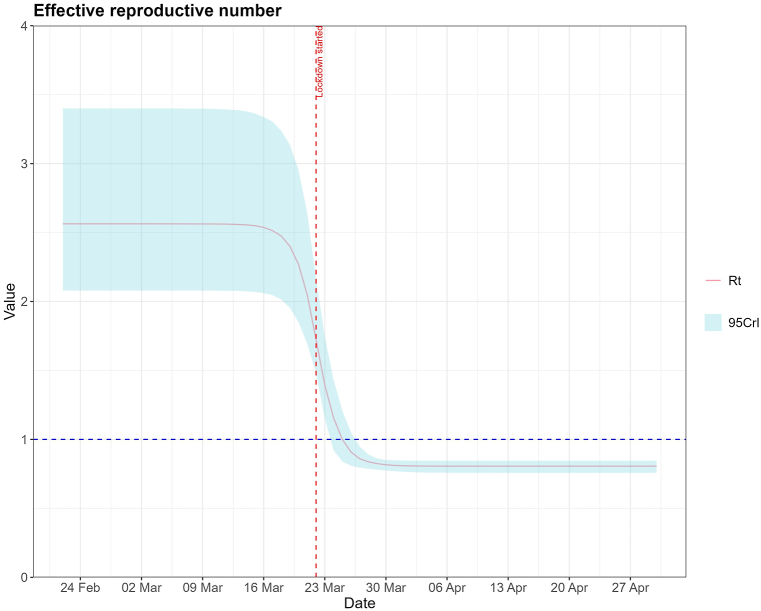
Rt for the Portuguese COVID-19 epidemic from 24th of February until 1st May.

**Fig. 2:**
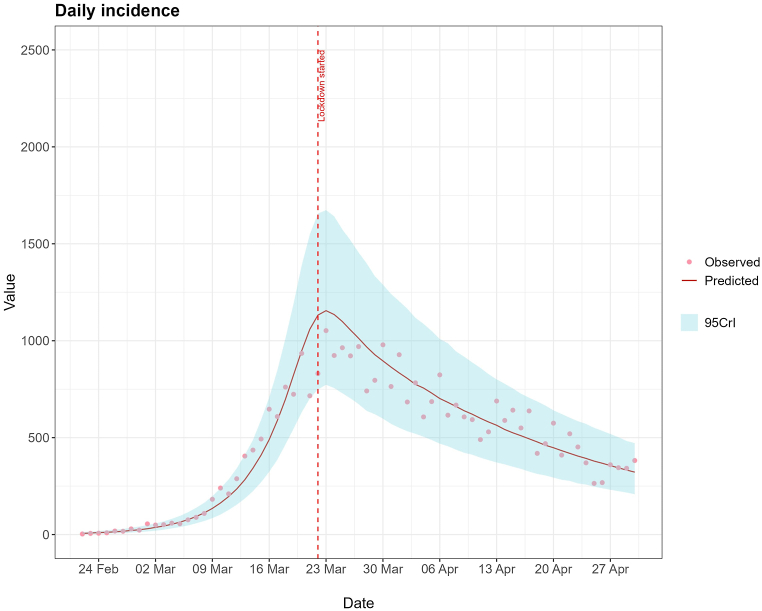
Observed number of COVID-19 cases and predictive posterior medians and 95%CrI given by the model.

The HMC algorithm was run through 4 Markov chains for 1000 warmup iterations and 4000 sampling iterations. Our total observed cases during the period of this study was 32879. The estimated parameters, fitting graphs, trace plots, and posterior distributions for each scenario are in the supplementary material. The results will discuss only the scenario with 36·6 % reporting probability, as it showed the best fit to observed data. The model fit to the data is displayed in Fig. 2. In the model, the lockdown final impact (*η*) had a median value of 0·314 (95%CrI: 0·225; 0·403). This means it reduced the overall transmission rate by 68·6 %. The reproductive number at each time point, affected by the NPIs, is shown in Fig. 1. The NPIs had an almost immediate effect, achieving 50 % of its impact almost instantly. On 24th February, the *R*_*t*_ was 2·62 (95%CrI: 2·11; 3·49) and was reduced to a minimum of 0·80 (95%CrI: 0·76; 0·84) shortly after the lockdown was introduced. Fig. 1 shows that the *R*_*t*_ decreased even before the NPI was introduced.

### 3.1 Counterfactual scenario

**Fig. 3:**
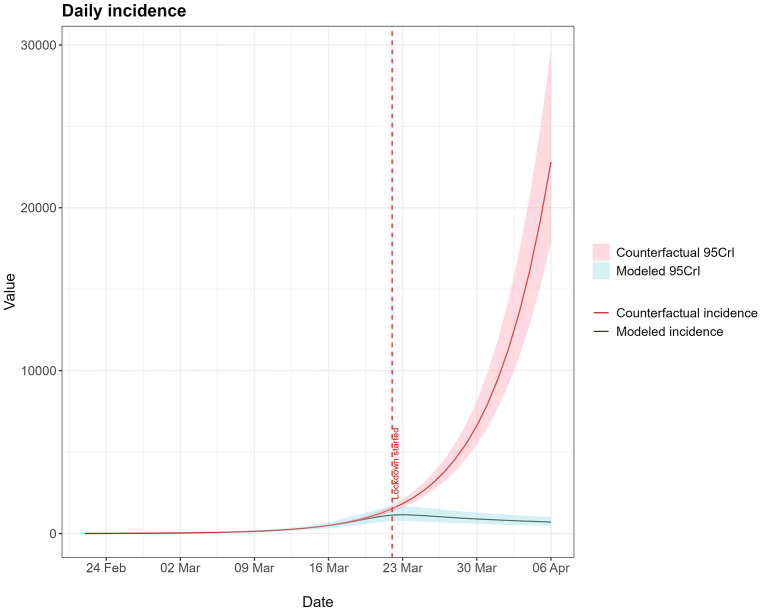
Modeled daily absolute incidence with NPI and counterfactual daily incidence during the 24th February and 6th April.

The counterfactual scenario, shown in Fig. 3, shows that the median modeled and counterfactual daily incidence (although not the 95%CrI) started diverging even before the lockdown was implemented. The total prevented cases were 118052 (95%CrI: 99464; 145605) from 24th February to 6th April.

## 4 Discussion

This study aimed to quantify the effect of NPIs on the transmission of COVID-19 in Portugal. While the effect of NPIs on lowering the transmission of COVID-19 in Portugal and other countries using compartmental models has already been thoroughly discussed in the scientific literature, this study quantified the impact of lockdown on the first COVID-19 wave in Portugal, as well as prevented cases, using Bayesian statistics with daily incidence.

The results show that the lockdown lowered *R*_*t*_ below 1, which means that on average, one individual infects less than one new individual, and the outbreak may be under control. As Fig. 1 shows, *R*_*t*_ started lowering even before the lockdown was in effect, which may be attributed to the placement of other NPIs, such as closing schools on 16th March [21] and some companies switching to hybrid or remote working. Still, *R*_*t*_ values aligned with published literature, which obtained similar values [3,10,17]. Both the incubation and recovery times were shorter than most articles suggest, with many proposing that COVID-19 has an incubation period of around 5 days [[22], [23], [24]] and a person remains infectious with COVID-19 for around 5–10 days [20]. However, some modelling studies infer an infectious period of 3 days as well [25,26]. The infectivity of a person may vary during this period, meaning that while a person is contagious for a more extended period, most transmissions occur in the first days [20]. The number of initially infectious and exposed individuals were about 42 and 26, respectively, which means that with a report probability of 36·6 %, only about 15 infectious would have been reported initially. Our sensitivity analysis supports this high number of initially infected individuals, as the models where a lower number of initially infected individuals was assumed, either had an unsatisfactory fit or produced a posterior distribution of initially infectious individuals closer to 42. The NPIs had a maximum reduction of 68·6 % in the transmission rate, resulting in a *R*_*t*_ below 1.

Other models that rely on simulations, such as fractional-order models have emerged as robust tools for understanding and managing disease dynamics. Recent studies highlight the utility of fractional-order systems in capturing the dynamics of disease transmission, such as memory effects and human behavior under NPIs [27,28]. They have been shown to robustly model disease spread while optimizing control measures [27,28]. As demonstrated in our study using real world data, the swift reduction in the reproductive number (Rt) due to NPIs is a tangible outcome of these interventions, but the results and NPIs may further be refined by recurring to other models that are more simulation-based, such as the ones using fractional-order systems.

The main downsides of NPIs, especially lockdowns, are the effects on the economy [29] and mental health of individuals [6]. Despite government and european aid to revitalize the economies, the economic costs of the NPIs are still high and the economy is still recovering [30]. However this downsides need to be compared with the benefits shown in this study, such as the prevented cases, which means less deaths and less burden on healthcare services, and also the reduction in *R*_*t*_ means that the pandemic was brought under control faster. When Portugal announced the nationwide lockdown, other european countries had already implemented it so there was a political precedent for it [31,32]. Regarding the closing of schools, the use of online and televised classes mitigated the impact on the education of children [33], although the effect of isolation may prove harder to solve [8]. Finally, the NPIs final impact heavily depends on adherence from the population which is heavily related with socioeconomic factors such as housing conditions, and education [34,35].

The first wave of the National Serological Survey, done around May 2020, indicates a seroprevalence of 2·9 % [36], further suggesting an underascertainment of COVID-19 cases during the first wave. However, this seroprevalence includes both symptomatic and asymptomatic individuals. It also shows that since only a small fraction of the population had been infected with COVID-19, the reduction in *R*_*t*_ could not be attributable to depletion of susceptibles.

Italy and Spain were also heavily affected by this first wave of covid-19 and would implement nationwide lockdowns too, on March 9 and March 14 respectively. The results in Italy are show that the *R*_*t*_ was already diminishing before lockdown, but nationwide lockdown was decisive in reducing it further below 1 [32,37]. In Spain, nationwide lockdown was also vital to reduce the *R*_*t*_ below 1, with one study suggesting an effectiveness of 68 % in reducing transmission [38], and another supporting a reduction in *R*_*t*_ from 5·89 before lockdown to 0·48 [31]. While Flaxman et al. produced similar effects to this model, showing an almost immediate reduction in the *R*_*t*_ in both Italy and Spain [14], studies using different methods, estimate that this reduction in *R*_*t*_ to below 1, happened not only over a large timespan, around 14 days, but also occurred differently between regions in each country [32,39]. The counterfactual scenario shows that if the contacts between individuals had not been reduced by implementing NPI, the number of cases would have been much greater and thus the overburdening of healthcare services and number of deaths. In fact, the Portuguese government acted quickly and avoided a much worse scenario during the first wave of COVID-19 [40]. The data used referred to a period where the disease was largely unknown and feared by the population, which may have increased adherence to the NPIs [34,35,41].

Regarding limitations, like all models, they represent an approximation of reality. The parameters used as priors are based on the available scientific literature, and although the examples most suited to the Portuguese reality are selected, it is impossible for them to be entirely identical or uniform for the studied population. The chosen parameters may depend on other variables not considered in this model, such as the immune status of each individual. Further research should focus on applying this methodology to other COVID-19 waves, such as a scenario where a vaccine is available or where housing conditions are different, to gain insight into the impact of NPIs such as lockdowns and also be able to apply it to other similar diseases. The motivation for this work stemmed largely from the need to gain deeper insights on how NPIs work to reduce contacts between people in the context of a transmissible disease. Flaxman et al. [14] provide the necessary methodology to achieve this. There was also access to better data that relies on symptom onset, which reduces the lag period present in hospitalization data, and minimizes batch reporting effect, and vastly published literature that provided valuable information to create the *priors* for the model. This way, a detailed and clear image about the impact of the NPIs on the portuguese population was able to be crafted in this study.

## 5 Conclusions

Our findings suggest that the NPIs were an effective measure to curb the transmission of epidemics. It produced effects quite fast and reduced the reproductive number of the first wave to under 1, which meant that incidence was in decline and disease spread may have been under control in a period before the deployment of COVID-19 vaccination. Since they can be applied to many infectious diseases that spread human-to-human, it is an effective, albeit costly, way to quickly reduce transmission and ease the load on hospitals and, ultimately, deaths. Thus, NPIs should be used in settings without other options, such as vaccination, or as a complement. This study may help other research being conducted in the mathematical modelling of infectious diseases by allowing the comparison of results between different territories, diseases, and NPIs. It should aid decision-makers in future scenarios where an NPI is considered to fight infectious disease, especially if it has transmission patterns similar to those of COVID-19.

## CRediT authorship contribution statement

**Dinis B. Loyens:** Writing – original draft, Visualization, Software, Investigation, Formal analysis. **Constantino Caetano:** Writing – review & editing, Validation, Software, Methodology, Data curation, Conceptualization. **Carlos Matias-Dias:** Supervision, Project administration.

## Data availability statement

The data used in this work may be available upon request and the fulfillment of certain criteria.

## Ethics declaration

This study was reviewed and approved by the Comissão de Ética para a Saúde do Instituto Nacional de Saúde Dr Ricardo Jorge (Health Ethics Committee of the National Health Institute of Portugal) with the approval number: 138, dated April 15, 2024.

## Funding

This research was supported by the Portuguese Government. No grant or extra funding was necessary.

## Declaration of competing interest

The authors declare that they have no known competing financial interests or personal relationships that could have appeared to influence the work reported in this paper.
