# Supplementary material for: Impact of non-pharmacological interventions on the first wave of COVID-19 in Portugal 2020
Source: Heliyon. 2025 Jan 31;11(4):e41569. doi: 10.1016/j.heliyon.2024.e41569 (PMC11868933; doi:10.1016/j.heliyon.2024.e41569)
Supplement: Multimedia component 1 [file mmc1.pdf]

Supplementary material to the "Impact of Non-Pharmacological Interventions on the first wave of Covid-19 in Portugal 2020"

September 19, 2024

1 Introduction

In this document, the methods, results, and sensitivity analysis will be shown in greater detail.

2 Fitting the model

The methodology proposed by Grinzstajn et al. [1] served as the basis for the analysis in this paper. A compartmental SEIR (Susceptible, Exposed, Infectious, and Removed) model was used to simulate the first COVID-19 wave in Portugal in 2020:

$$\frac{dS(t)}{dt} = -\beta(t)\frac{S(t)I(t)}{N} \tag{1}$$

$$\frac{dE(t)}{dt} = \beta(t)\frac{S(t)I(t)}{N} - \alpha E(t) \tag{2}$$

$$\frac{dI(t)}{dt} = \alpha E(t) - \gamma I(t) \tag{3}$$

$$\frac{dR(t)}{dt} = \gamma I(t) \tag{4}$$

Table 1 describes the variables and parameters used in our model. The time unit for this model is the day. The progression from exposed to infectious and recovery rates were obtained by calculating the inverse of the incubation and infectious periods, respectively. Flaxman *et al.* [2] developed a function that describes the effect of each NPI on the transmission rate of a disease:

$$g(t) = \eta + (1-\eta)\cdot\frac{1}{1+e^{\xi(t-t_1-\nu)}} \tag{5}$$

Thus, β and the effective reproductive number, R_t, vary in time. β can now be defined as $\beta = \beta \cdot g(t)$ and $R_t = \frac{\beta}{\gamma} \cdot g(t) \cdot \frac{S}{N}$. $g(t)$ may only take values between 0 and 1, with 1 meaning that the NPI had no effect on transmission, and 0 meaning that the NPI reduced transmission to 0.

Table 1: Variables and parameters used

Variable	Description
S	Susceptible individuals
E	Exposed individuals
I	Infectious individuals
R	Removed individuals
β	Transmission rate
α	Progression from exposed to infectious rate
γ	Recovery rate
η	Final impact of the NPI
ν	The time between the start of the NPI and achieving 50% of its total effect
t_1	NPI starts having effect
ξ	The slope of the transmission variation
μ_t	Daily absolute incidence

The t_1 (described on table 1) used in the model was 22nd March 2020, as it was the date that the lockdown started [3]. The data used for this study was the daily absolute incidence in Portugal between the 22nd of February and the 1st of May 2020, based on the date of symptom onset. This data was extracted from the Portuguese notifiable disease database (BI-SINAVE). Population data was obtained from the Portuguese National Statistics. Our model was fitted to observed data by daily absolute incidence, defined by the following equation

$$\mu_t = (E(t) - E(t+1) + S(t) - S(t+1)) \times \%\text{reported} \tag{6}$$

Due to scarcity of covid-19 tests available and testing policies [4], there was an under-ascertainment of real cases among the population. According to the literature, the reporting probability of a case was between 22% and 36.6% [5].

Table 2: Priors used in the study

Prior	Distribution		Reference
β	Normal(mean=2, SD=1)		Grinzstan *et al.*[1]
γ	Gamma(mean=4,var=20)		Byrne *et al.*[6]
α	Normal(mean=0.3, SD=0.001)		Byrne *et al.*[6]
$\frac{1}{\phi}$	Exponential(rate=5)		Assumed
The initial number of infectious (i0)	Normal(mean=1, SD=10)	Normal(mean=1, SD=2)	Assumed
The initial number of exposed (e0)	Normal(mean=1, SD=10)	Normal(mean=1, SD=2)	Assumed
η	Beta($\alpha = 4$, $\beta = 8$)		Assumed
ν	Exponential(rate=1/2)		Assumed
ξ	Beta($\alpha = 1$, $\beta = 1$)		Assumed

SD - Standard Deviation; var - variance

A sensitivity analysis on the importance of the %report parameter was performed, to ensure which values better fit the observed data and reality, since the true reporting probability is unknown. The model was calibrated in 8 scenarios: if the initial infected followed a normal distribution of mean 1 and standard deviation 10 and a normal distribution with mean 1 and standard deviation 2. These assumptions were run four times, assuming values of report probability taken from the literature: of 22%; 36.6%; as a prior with a distribution of Beta(α=8;β=14), which has a mean of 0.36 and a variance of 0.01; and 100%. The projected daily incidence without the NPIs was calculated with a counterfactual scenario, thus allowing the calculation of prevented cases by the NPI during the first 15 days of its effect. Table 3 summarises the assumptions in each scenario.

Table 3: Scenarios used for the sensitivity analysis

Scenario	Initial infectious and exposed individuals prior distribution	Percentage of cases reported
1	Normal (mean=1, SD=10)	22%
2	Normal (mean=1, SD=10)	36.6%
3	Normal (mean=,1 SD=10)	Beta(α=8; β=14)
4	Normal (mean=1, SD=10)	100%
5	Normal (mean=1, SD=2)	22%
6	Normal (mean=1, SD=2)	36.6%
7	Normal (mean=1, SD=2)	Beta(α=8; β=14)
8	Normal (mean=1, SD=2)	100%

The posterior distributions were obtained using Markov Chain Monte Carlo (MCMC) algorithms, particularly Hamilton Monte Carlo (HMC). The model was calibrated using R, specifically R version 4.3.3 and the package rstan version 2.32.5. The model was written in STAN. A 95% Credible Interval (95%CrI) was obtained for each prior distribution. For the posterior distributions in each scenario, the mean, standard error (se_mean), standard deviation (sd), 2.5%, 25%, 50%, 75% and 97.5% percentiles, the effective sample size and the Rhat will be shown.

3 Results

The HMC algorithm was run through 4 Markov chains for 1000 warmup iterations and 4000 sampling iterations. After running all 8 scenarios, only scenario 2 was presented in the article as it was the one that had the best fit to observed data, and the reporting probability was validated in literature [5].

4 Scenario 1

Scenario 1 had a report probability of 22% and an initial infectious group prior of Normal (1, 10). After running the algorithm with these priors, we obtained the results shown in Table 4. The modelled data fit to observed data is shown in Figure 1, and the variation in time of R_t is shown in Figure 2. The chains have mixed well as shown by the $Rhat \leq 1.01$ and the trace plots in Figure 3. This fit has more uncertainty than scenario 2.

Table 4: Posterior distributions for Scenario 1

Parameter	Mean	SE Mean	SD	2.5%	25%	50%	75%	97.5%	n_{eff}	Rhat
β	1.125	0.003	0.241	0.735	0.949	1.098	1.275	1.660	5771.696	1.001
γ	0.494	0.002	0.153	0.242	0.381	0.477	0.591	0.829	5791.299	1.001
η	0.353	0.001	0.045	0.255	0.324	0.356	0.386	0.427	6011.081	1.001
ξ	0.866	0.002	0.283	0.511	0.620	0.798	1.080	1.451	14621.370	1.000
ν	0.096	0.001	0.096	0.002	0.028	0.067	0.133	0.355	15340.620	1.000
R_0	2.356	0.004	0.277	1.986	2.158	2.303	2.489	3.041	5882.708	1.001
Recovery time	2.242	0.010	0.768	1.206	1.693	2.097	2.621	4.133	5737.711	1.001
Incubation time	3.333	0.000	0.011	3.311	3.325	3.333	3.340	3.354	17722.590	1.000
Initial infectious	52.206	0.074	7.274	38.039	47.307	52.094	57.101	66.633	9779.595	1.000
Initial exposed	31.449	0.095	9.166	13.357	25.296	31.409	37.531	49.551	9359.304	1.000
ϕ	23.328	0.048	5.278	14.344	19.609	22.829	26.537	35.042	12213.130	1.000

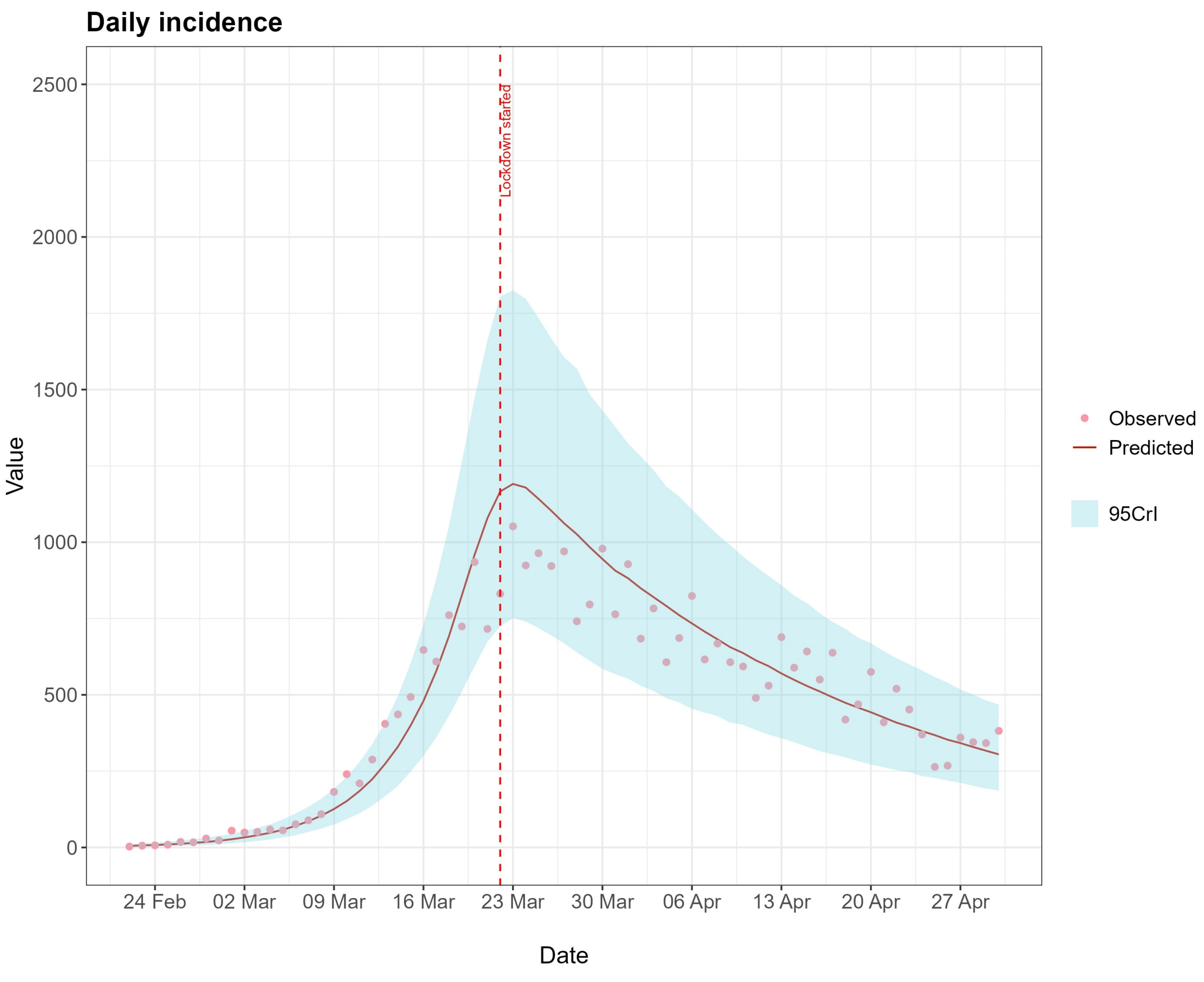

Figure 1: Observed number of COVID-19 cases and predictive posterior medians and 95% credible intervals given by the model for Scenario 1

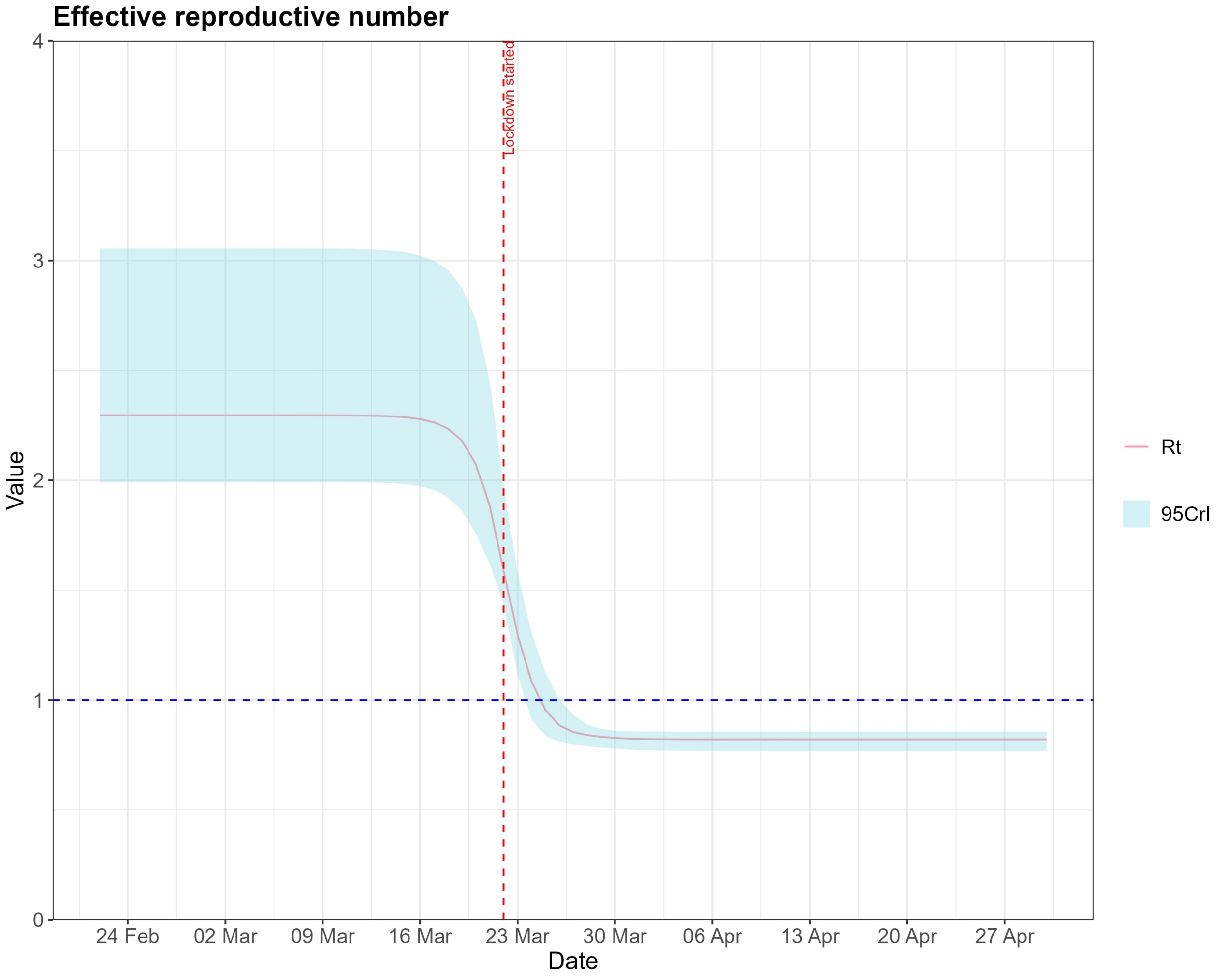

Figure 2: R_t for the Portuguese COVID-19 epidemic from 24th of February until May 1st for Scenario 1

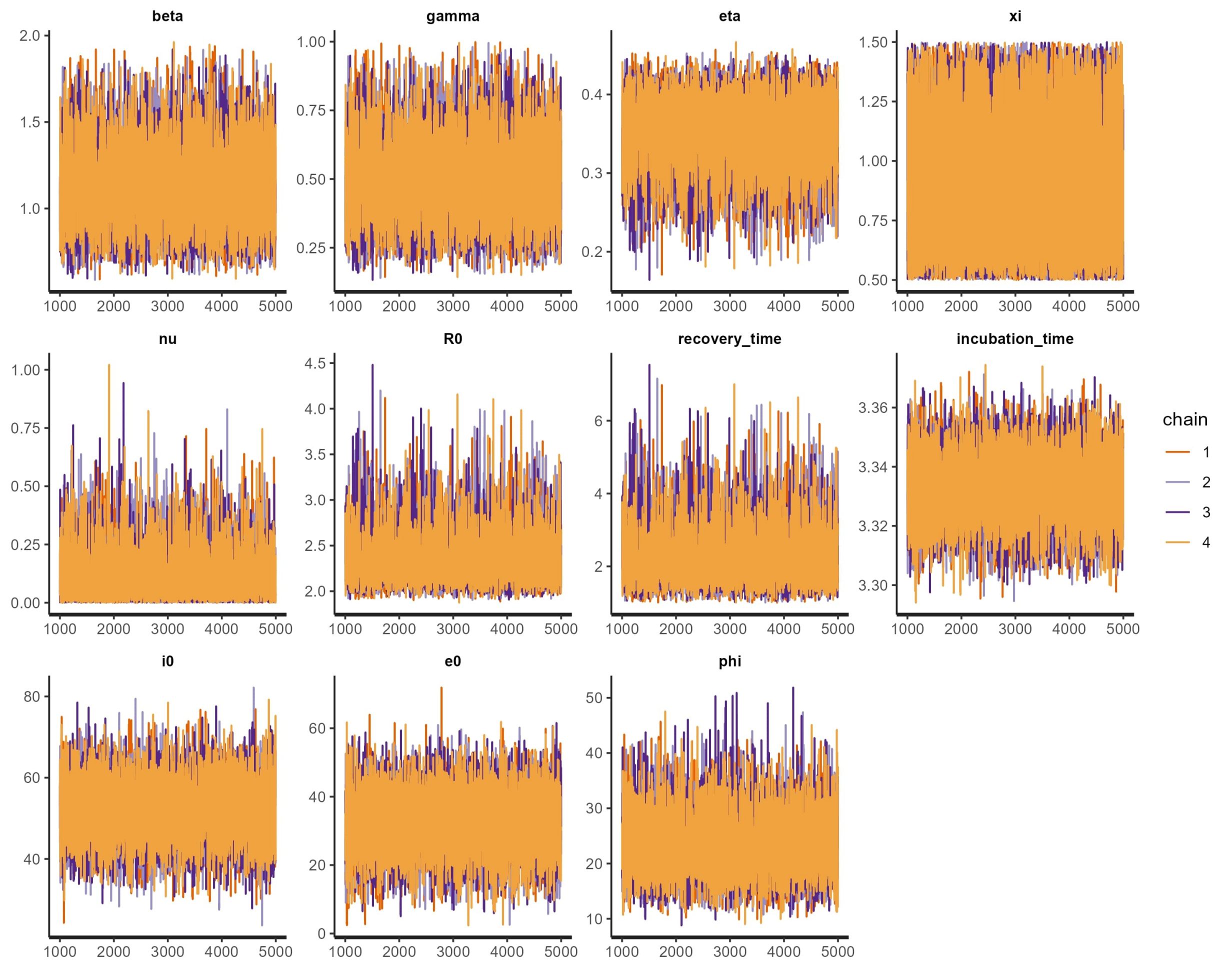

Figure 3: Posterior parameter trace plots with 4 chains and 4000 samples in Scenario 1

5 Scenario 2

Scenario 2 had a report probability of 36.6% and a prior for the initially infectious and exposed individuals following a Normal distribution with mean 1 and standard deviation 10. After running the algorithm with these priors, we obtained the results shown in Table 5. Although the number for initially infected and exposed individuals are quite high, this scenario showed the best fit to observed data, as shown in figure 4 and thus was chosen for discussion in the main article. The R_t is shown in figure 5. The traceplot (figure 6) and Rhat values show that the chains have mixed well.

Table 5: Posterior distributions for Scenario 2

	Mean	SE Mean	SD	2.5%	25%	50%	75%	97.5%	n_{eff}	Rhat
β	0.847	0.002	0.168	0.61	0.725	0.816	0.938	1.255	4575.618	1.000
γ	0.338	0.002	0.109	0.181	0.258	0.318	0.398	0.600	4592.577	1.000
η	0.314	0.001	0.047	0.225	0.281	0.314	0.347	0.406	5028.726	1.000
ξ	0.808	0.003	0.266	0.508	0.590	0.724	0.973	1.434	10771.460	1.000
ν	0.080	0.001	0.110	0.003	0.034	0.079	0.152	0.406	14824.210	1.000
R_0	2.610	0.005	0.344	2.083	2.355	2.564	2.816	3.400	5321.825	1.000
Recovery time	3.263	0.014	1.012	1.666	2.510	3.142	3.881	5.534	5203.577	1.000
Incubation time	3.333	0.000	0.011	3.311	3.325	3.333	3.341	3.355	16159.810	1.000
$i0$	42.766	0.074	6.758	29.637	38.168	42.746	47.357	56.118	8335.383	1.000
$e0$	24.911	0.095	8.750	7.740	18.867	24.913	30.861	42.036	8492.000	1.000
ϕ	30.175	0.062	6.807	18.708	25.317	29.521	34.366	45.256	12111.430	1.000

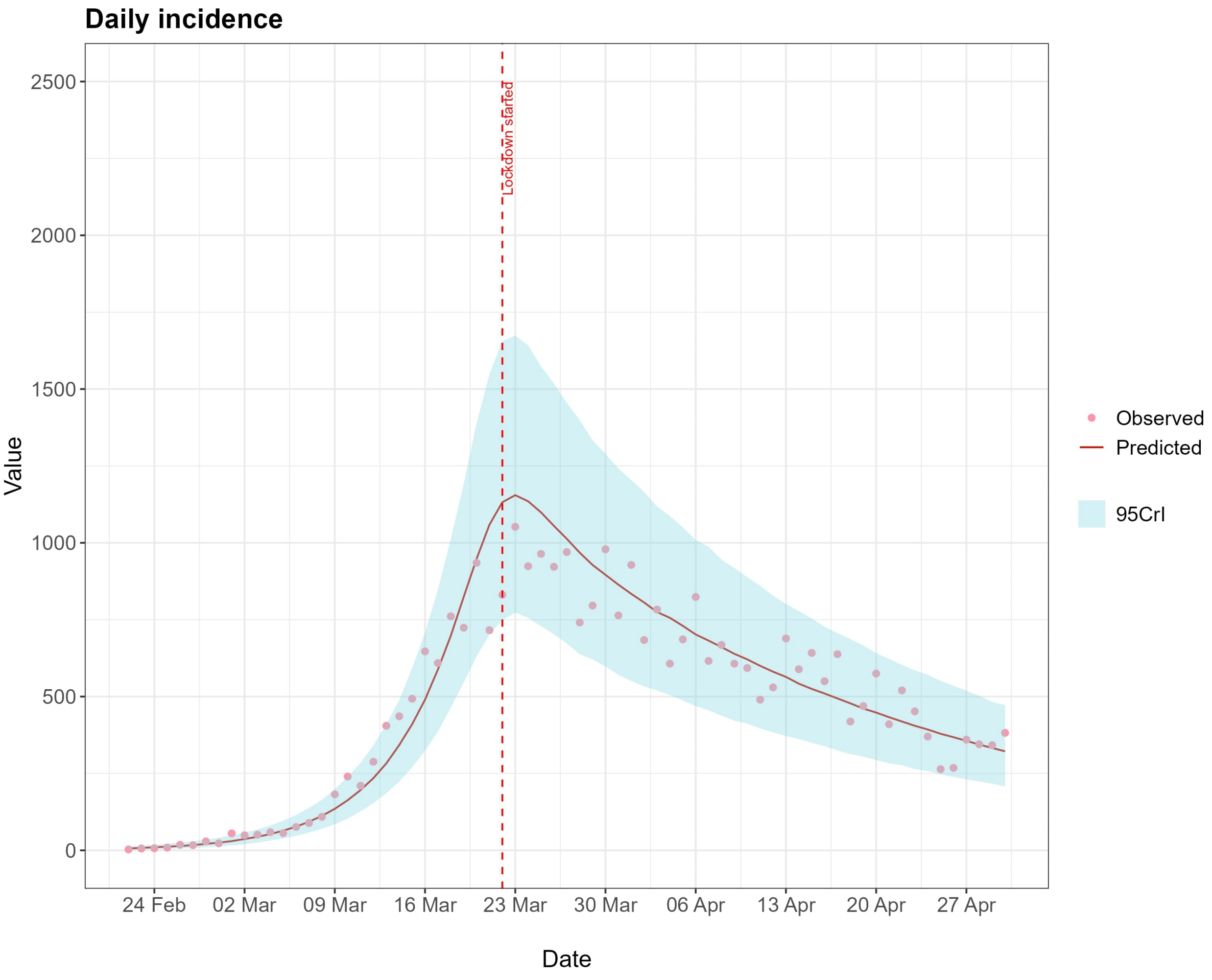

Figure 4: Observed and predicted daily absolute incidence during the study period for Scenario 2.

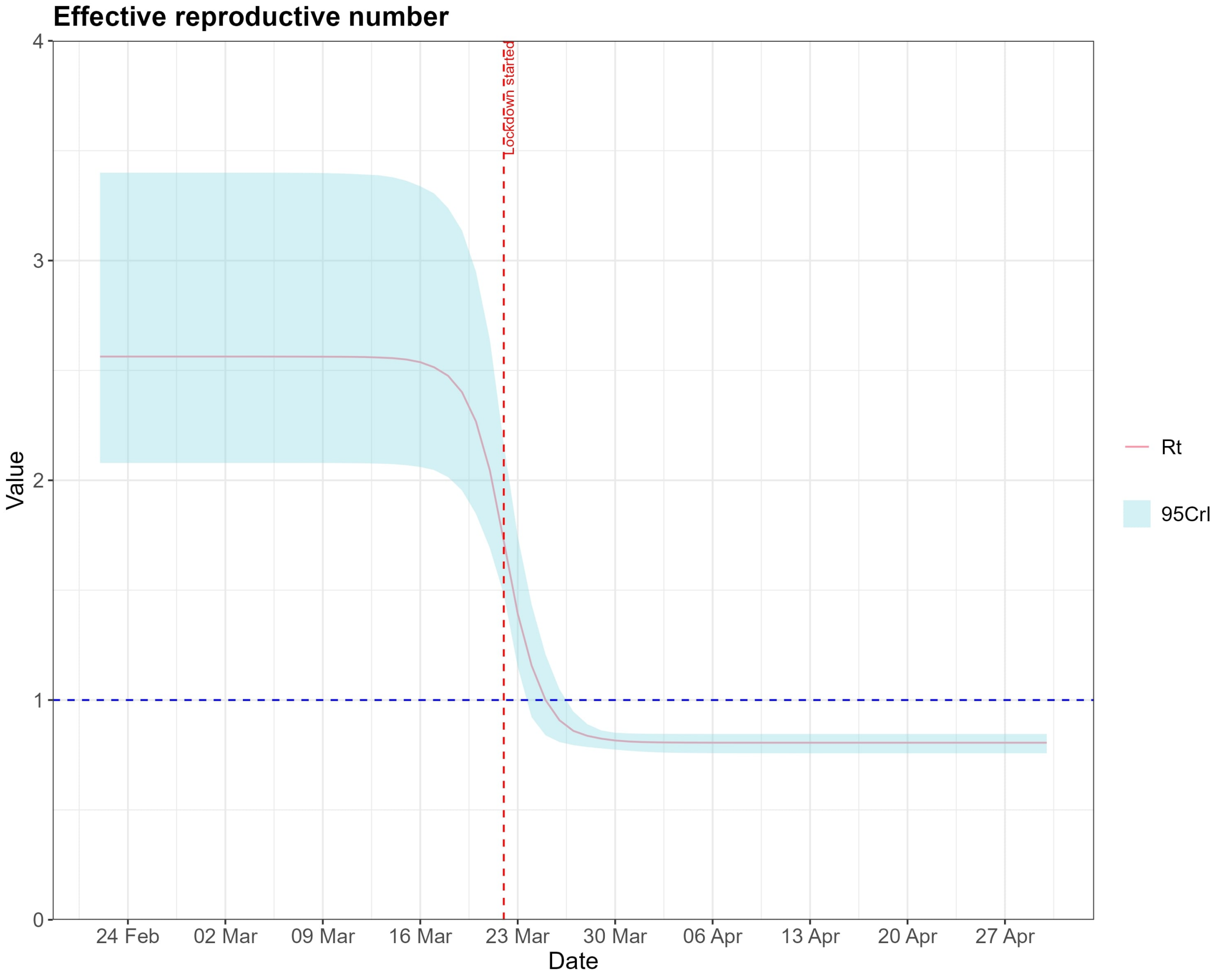

Figure 5: R_t for the Portuguese COVID-19 epidemic from 24th of February until May 1st for Scenario 2.

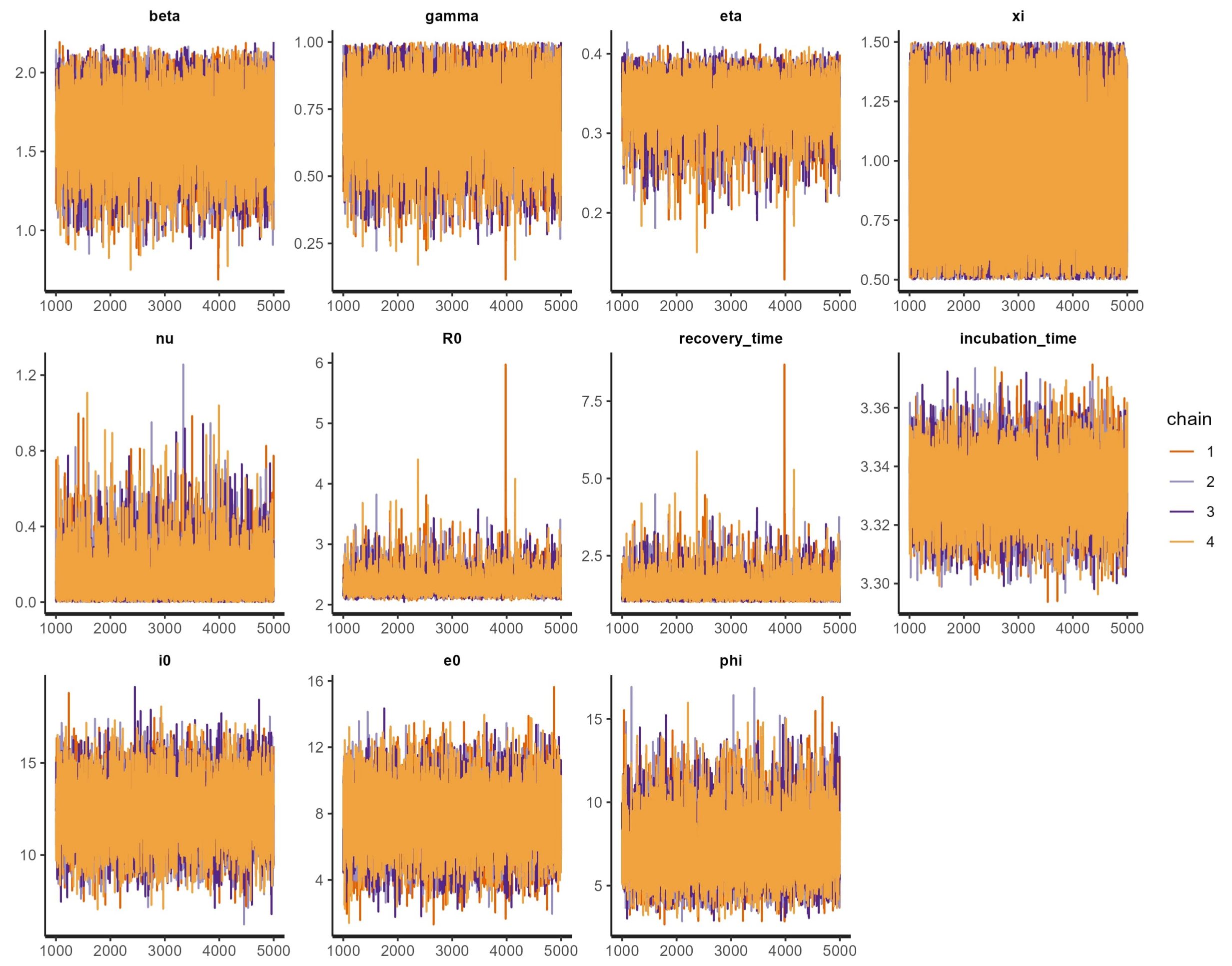

Figure 6: Posterior parameter trace plots with 4 chains and 4000 samples in Scenario 2

6 Scenario 3

In this scenario, the prior for initially exposed and infectious individuals remained with a distribution of Normal (1;10) however the reporting probability was considered a prior too with a distribution of Beta (α=8; β=14). The results were similar to Scenario 2, as seen on Table 6, and also the model fit to observed data (figure 7). The R_t during the studied period is shown in figure 8. It is noteworthy, in this scenario, that the modeled reporting probability was very close to 36.6%.

Table 6: Posterior distributions for Scenario 3

	Mean	SE Mean	SD	2.5%	25%	50%	75%	97.5%	n_{eff}	Rhat
β	0.842	0.002	0.165	0.605	0.723	0.812	0.932	1.229	5973.664	1.000
γ	0.335	0.001	0.107	0.178	0.257	0.316	0.394	0.588	6001.940	1.000
η	0.313	0.001	0.046	0.224	0.281	0.313	0.346	0.402	6762.809	1.000
ξ	0.807	0.002	0.267	0.508	0.589	0.722	0.973	1.431	12612.900	1.000
ν	0.112	0.001	0.109	0.003	0.032	0.078	0.157	0.402	16791.650	1.000
R_0	2.616	0.004	0.346	2.090	2.363	2.568	2.817	3.425	7181.473	1.000
Recovery time	3.288	0.012	1.020	1.700	2.540	3.164	3.894	5.624	7026.259	1.000
Incubation time	3.333	0.000	0.011	3.311	3.325	3.333	3.340	3.355	20952.540	1.000
i_0	42.385	0.068	6.779	29.099	37.877	42.339	46.930	55.841	10058.510	1.000
e_0	24.811	0.095	8.795	7.706	18.803	24.769	30.750	42.148	8628.264	1.000
ϕ	30.306	0.059	6.759	18.854	25.458	29.682	34.528	45.121	13025.740	1.000
% Report	0.372	0.000	0.010	0.352	0.365	0.372	0.378	0.392	15832.300	1.000

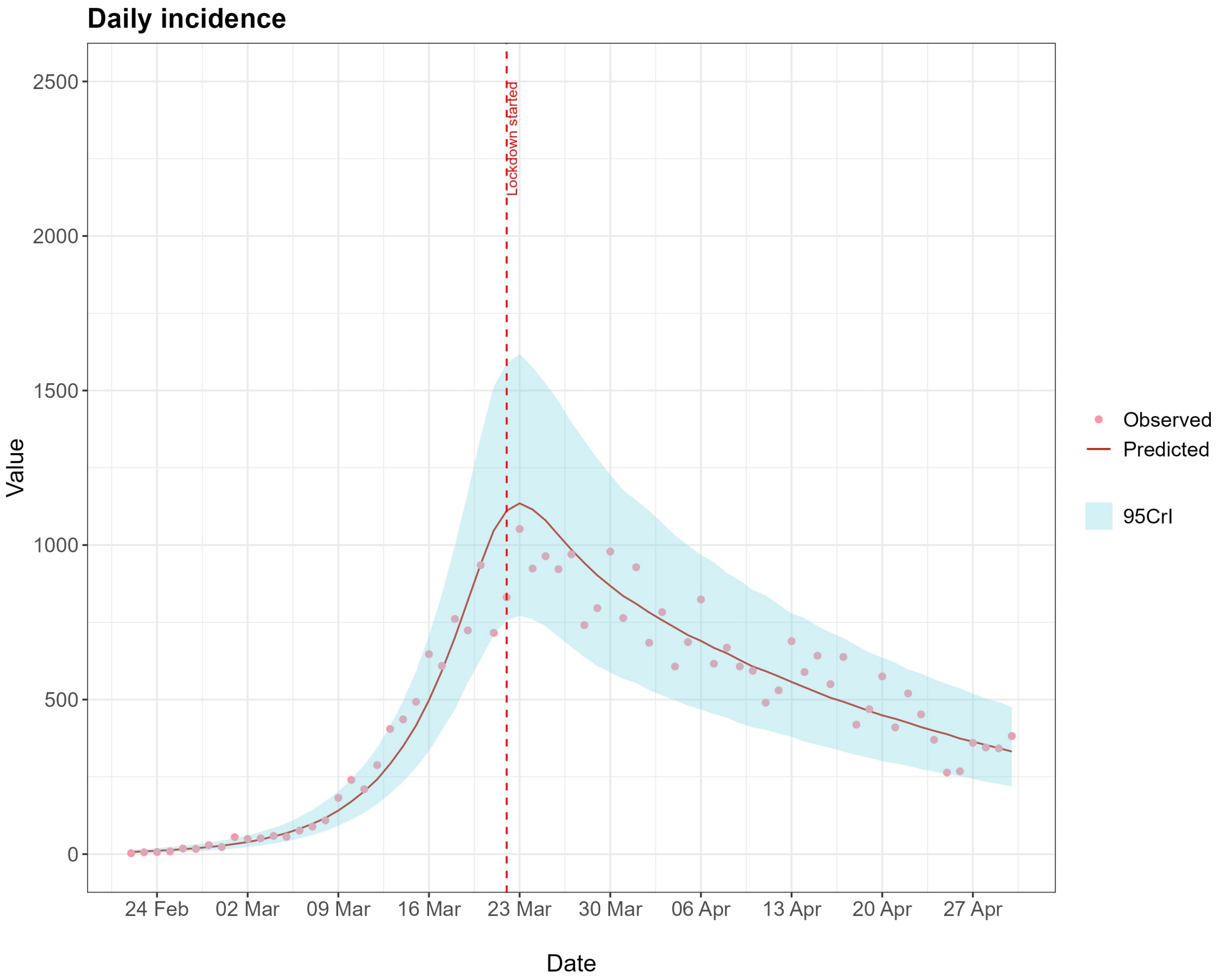

Figure 7: Observed and predicted daily absolute incidence during the study period for Scenario 3.

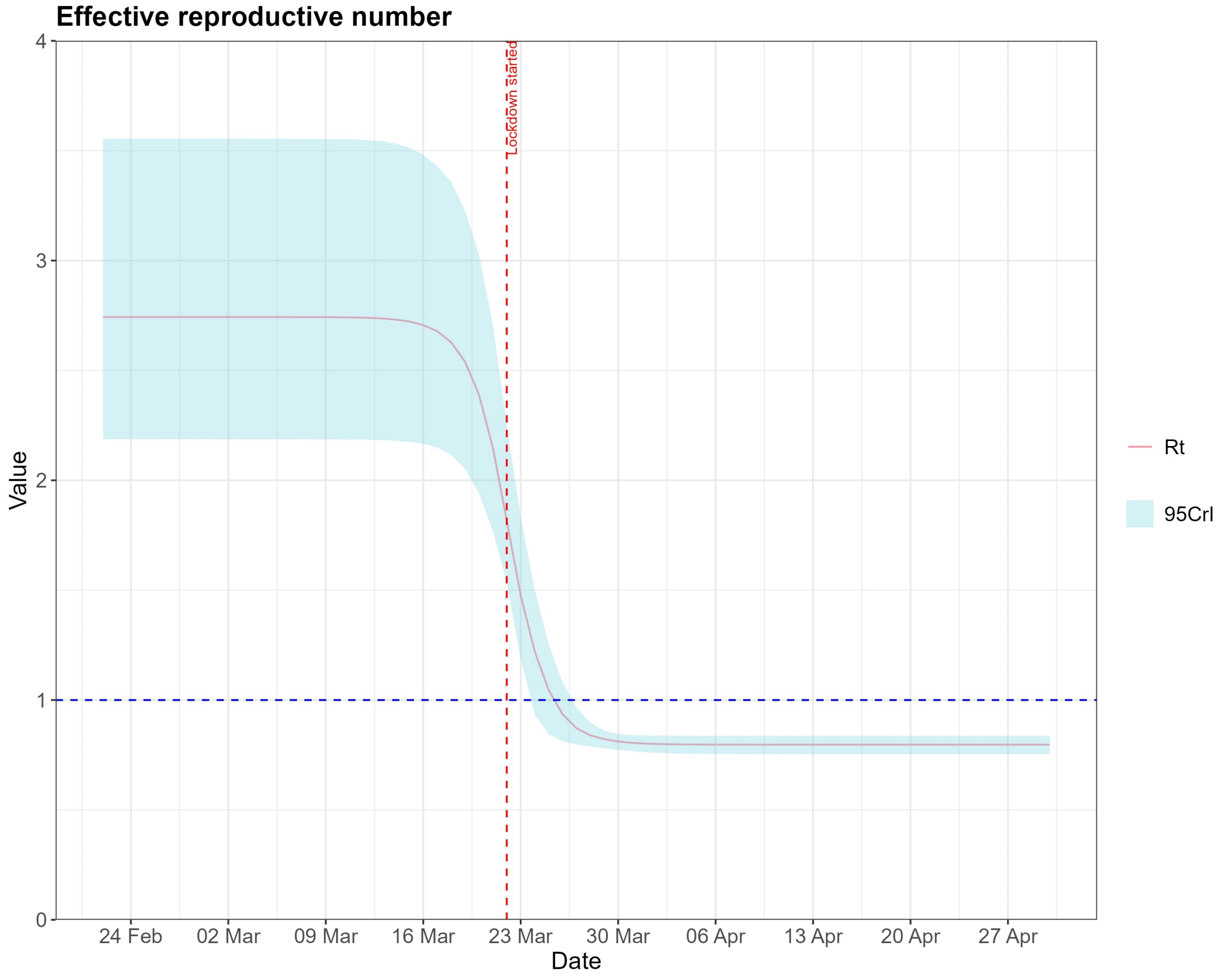

Figure 8: R_t for the Portuguese COVID-19 epidemic from 24th of February until May 1st for Scenario 3.

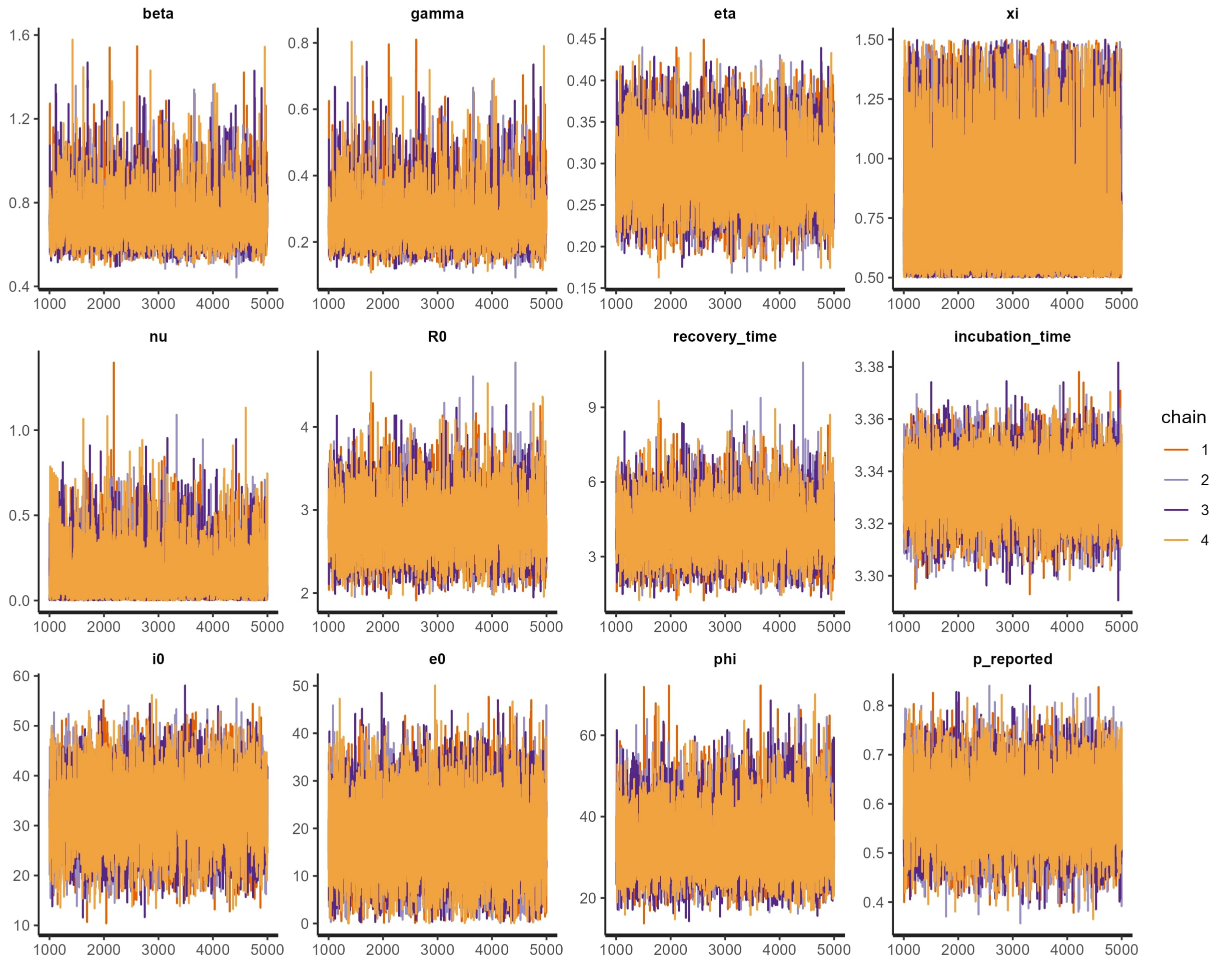

Figure 9: Posterior parameter trace plots with 4 chains and 4000 samples in Scenario 3.

7 Scenario 4

In this scenario, the report probability was 100% which means that there was no ascertainment bias. The main changes were essentially to the number of initially exposed and infectious individuals. While the β was slightly lower, the R_0 remained the same. The Rhat values and the traceplots show that the chains have mixed well. This scenario was not chosen however because it does not take into account the underascertainment of cases which is known to have existed and thus might not be an adequate representation of reality as other scenarios. [5]

Table 7: Posterior distributions for Scenario 4.

	mean	se_mean	sd	2.5%	25%	50%	75%	97.5%	n_eff	Rhat
β	0.677	0.002	0.097	0.536	0.609	0.661	0.726	0.913	4046.3	1
γ	0.245	0.001	0.064	0.151	0.200	0.234	0.277	0.399	4000.776	1
η	0.283	0.001	0.038	0.213	0.257	0.281	0.308	0.363	4431.011	1
ξ	0.721	0.002	0.223	0.505	0.556	0.640	0.812	1.340	8948.573	1
ν	0.164	0.001	0.158	0.004	0.049	0.116	0.228	0.588	14461.390	1
R_0	2.847	0.005	0.336	2.271	2.610	2.819	3.051	3.591	4757.235	1
recovery time	4.347	0.016	1.061	2.506	3.606	4.267	5.006	6.640	4634.337	1
incubation time	3.333	0.000	0.011	3.312	3.326	3.333	3.341	3.355	15688.220	1
i0	22.017	0.057	4.936	11.914	18.683	22.298	25.504	31.085	7423.729	1
e0	10.495	0.074	6.568	0.655	5.299	9.676	14.780	25.038	7836.867	1
ϕ	35.454	0.075	7.720	22.332	29.944	34.778	40.220	52.160	10456.510	1

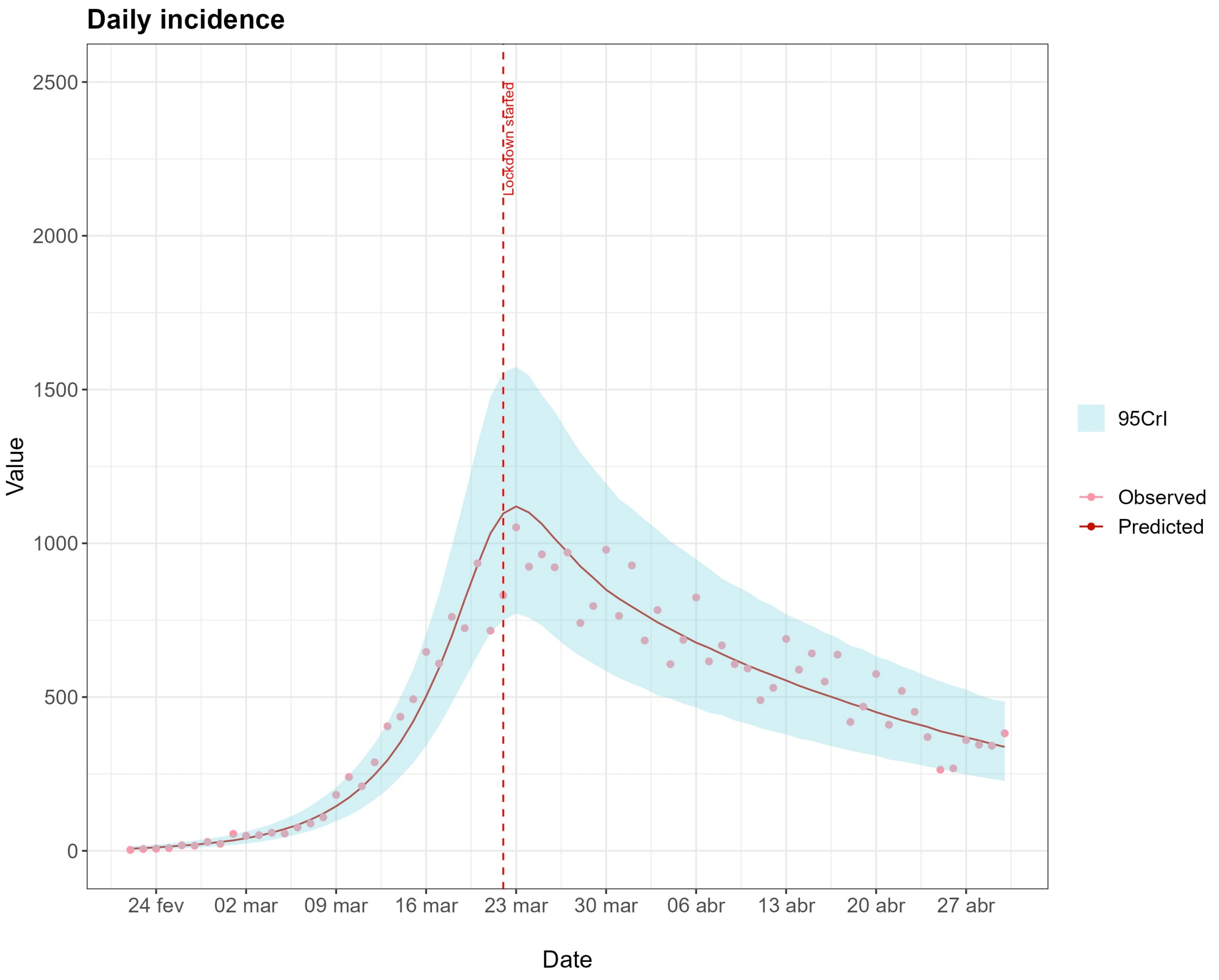

Figure 10: Observed and predicted daily absolute incidence during the study period for Scenario 4.

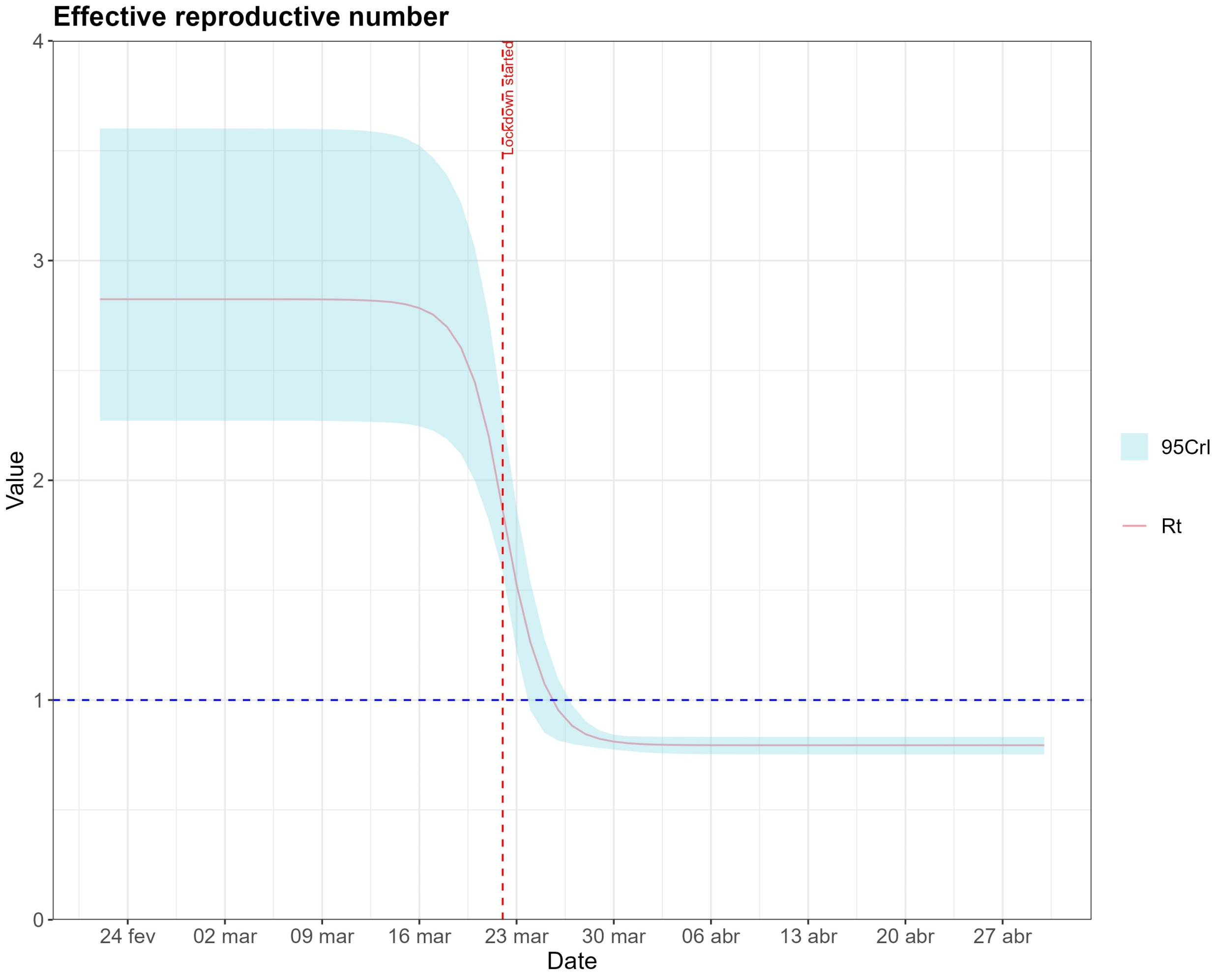

Figure 11: R_t for the Portuguese COVID-19 epidemic from 24th of February until May 1st for Scenario 4.

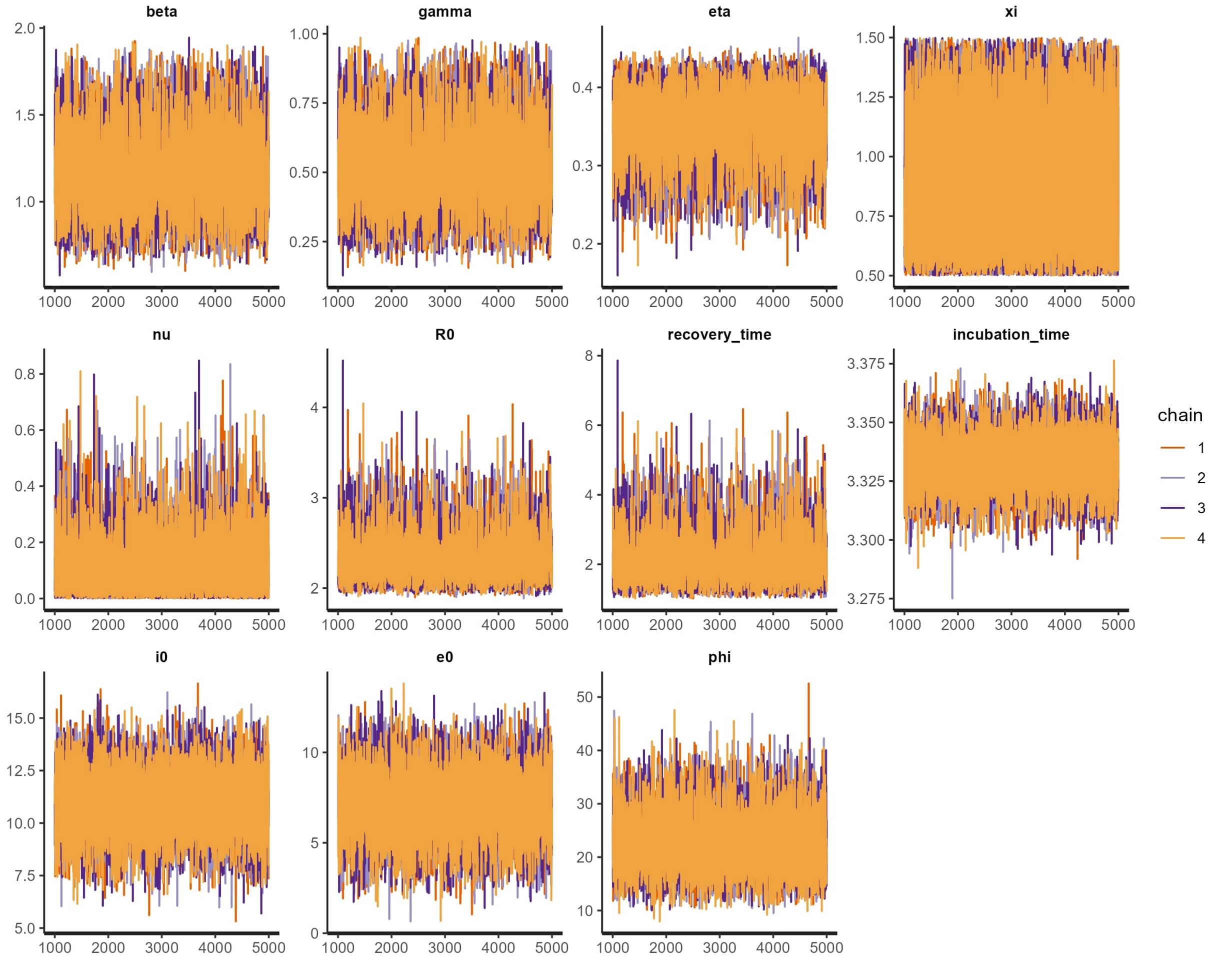

Figure 12: Posterior parameter trace plots with 4 chains and 4000 samples in Scenario 4

8 Scenario 5

In this scenario, the i0 and e0 priors were a normal distribution with mean 1 and standard deviation 2 but the reporting probability was assumed to be 22%. As seen in figure 13, the model fit is not as satisfactory as other scenarios, since the observed data is consistently below the median estimated values.

Table 8:

	mean	se_mean	sd	2.5%	25%	50%	75%	97.5%	n_eff	Rhat
β	1.624	0.004	0.256	1.116	1.443	1.628	1.816	2.074	4778.101	1
γ	0.7	0.002	0.154	0.395	0.592	0.703	0.815	0.97	4659.052	1
η	0.337	0	0.033	0.26	0.318	0.341	0.361	0.387	4517.627	1
ξ	0.93	0.003	0.3	0.512	0.659	0.894	1.184	1.469	13082.7	1
ν	0.117	0.001	0.119	0.003	0.033	0.08	0.162	0.434	15949.31	1
R_0	2.36	0.003	0.201	2.122	2.225	2.317	2.444	2.851	3987.971	1
recovery_time	1.514	0.007	0.417	1.031	1.227	1.422	1.69	2.533	4104.463	1
incubation time	3.332	0	0.011	3.311	3.325	3.332	3.34	3.354	17836.61	1
i0	12.342	0.017	1.613	9.162	11.254	12.347	13.429	15.491	9331.486	1
e0	7.525	0.019	1.887	3.906	6.21	7.519	8.836	11.266	10189.98	1
ϕ	7.294	0.021	1.849	4.256	5.975	7.103	8.392	11.522	8077.164	1

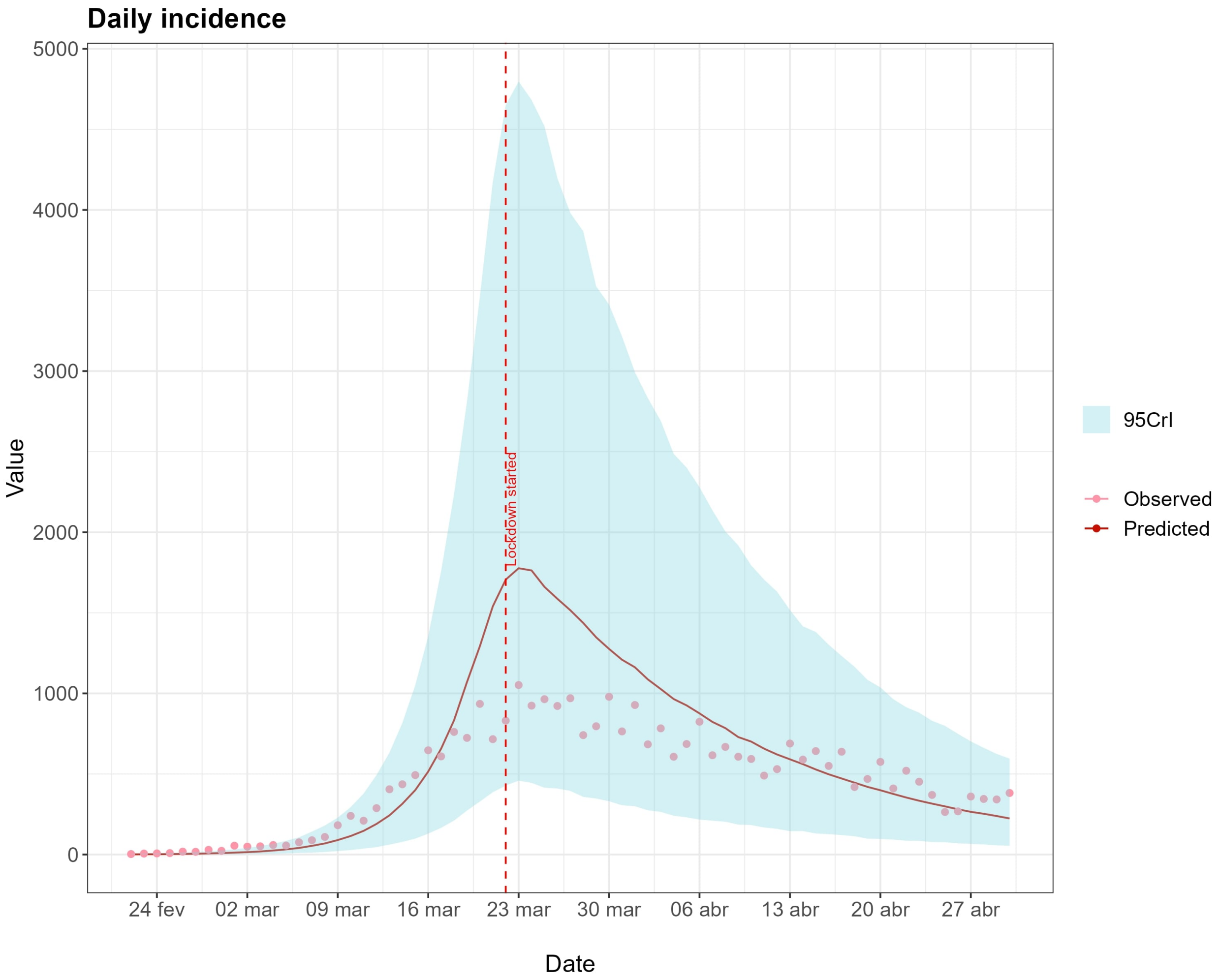

Figure 13: Observed and predicted daily absolute incidence during the study period for Scenario 5.

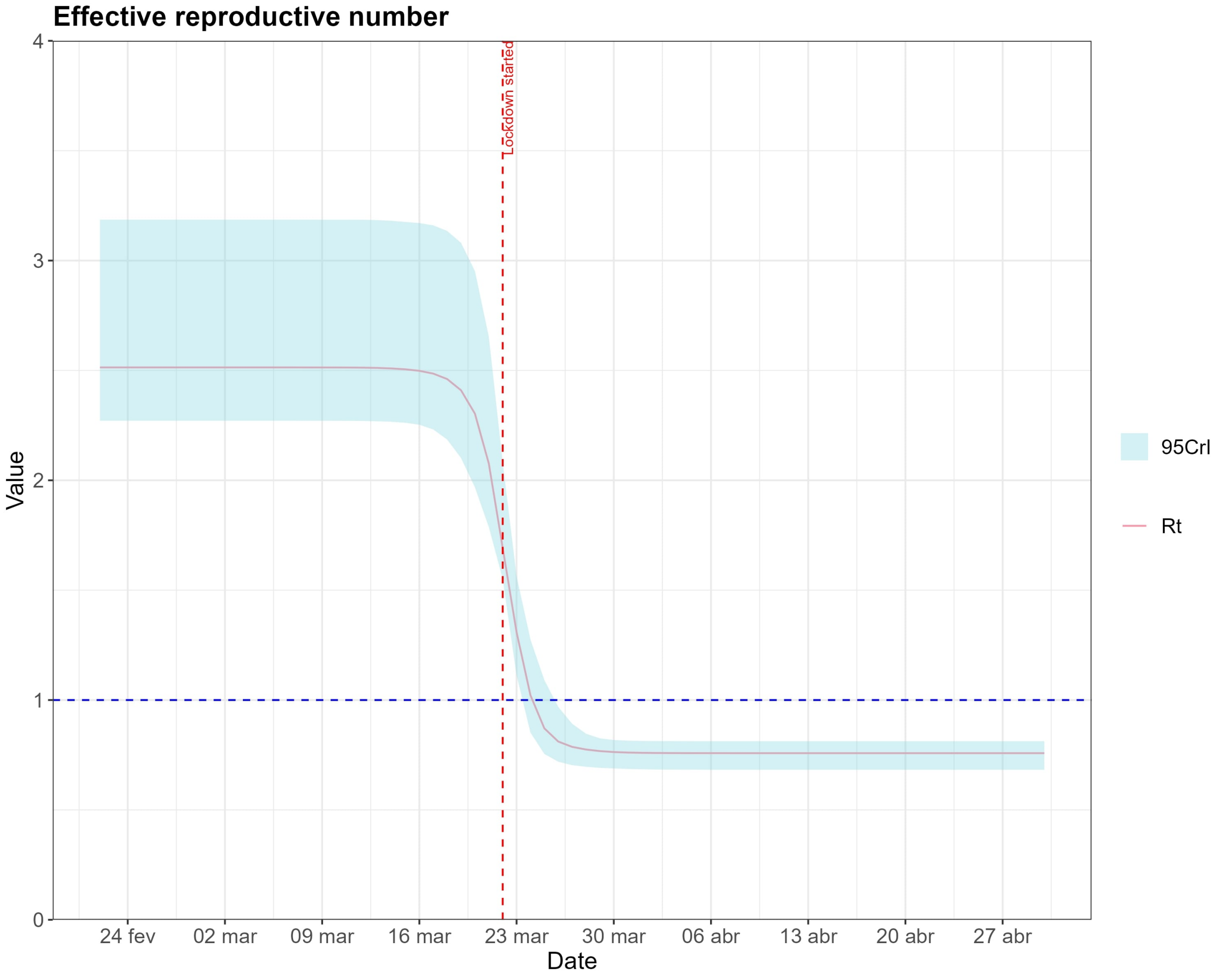

Figure 14: R_t for the Portuguese COVID-19 epidemic from 24th of February until May 1st for Scenario 5.

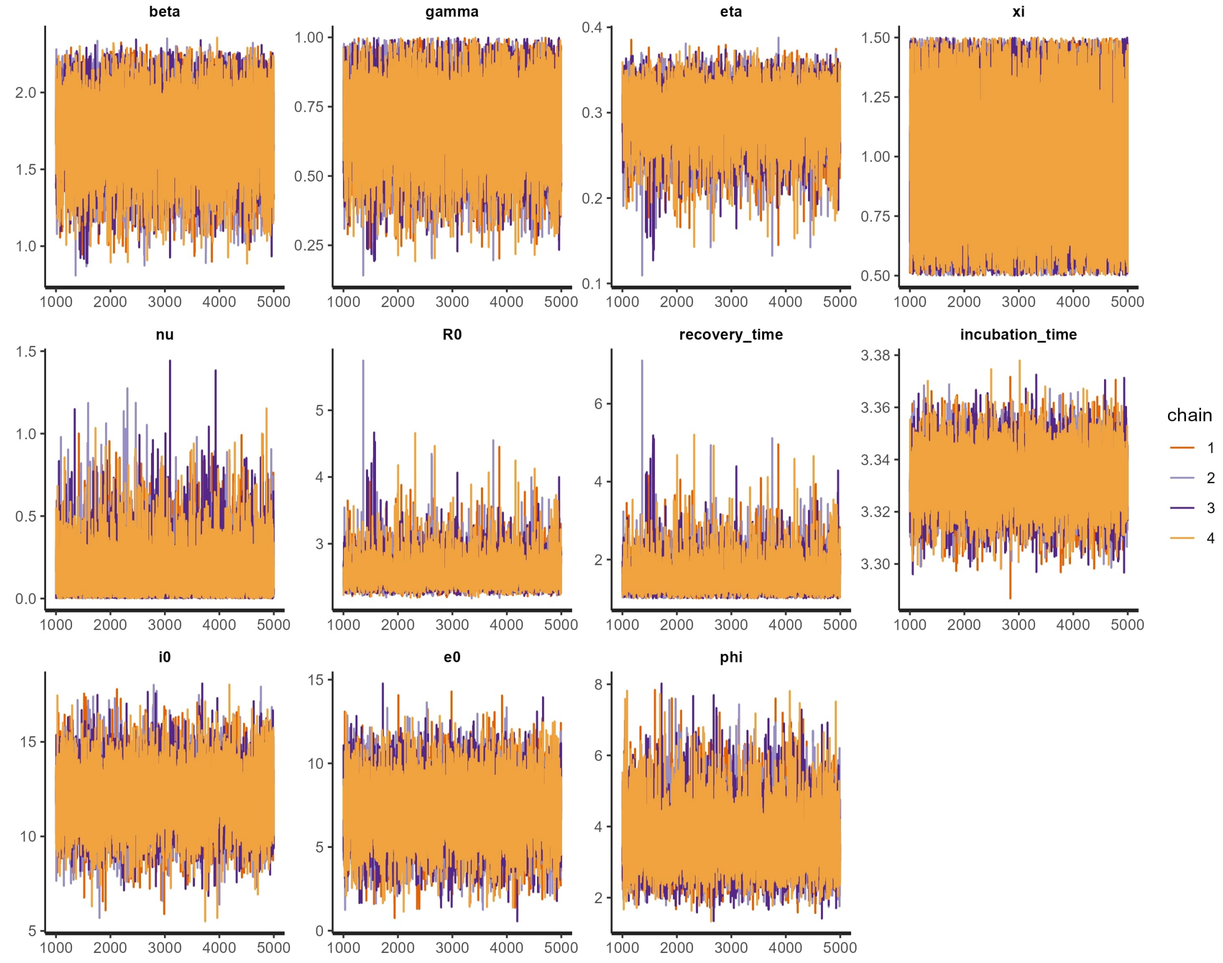

Figure 15: Posterior parameter trace plots with 4 chains and 4000 samples in Scenario 5

9 Scenario 6

As with scenario 5. the i0 and e0 had a more restrictive prior and while we can see that the chains have mixed well (Rhat in table 8 and figure 15), the observed data was consistently below the estimated median values, thus not being as satisfactory as other scenarios. The transmission rate (β) was much higher.

Table 9: Posterior distributions for Scenario 6

	mean	se_mean	sd	2.5%	25%	50%	75%	97.5%	n_eff	Rhat
β	1.624	0.004	0.256	1.116	1.443	1.628	1.816	2.074	4778.101	1
γ	0.700	0.002	0.154	0.395	0.592	0.703	0.815	0.970	4659.052	1
η	0.337	0.000	0.033	0.260	0.318	0.341	0.361	0.387	4517.627	1
ξ	0.930	0.003	0.300	0.512	0.659	0.894	1.184	1.469	13082.700	1
ν	0.117	0.001	0.119	0.003	0.033	0.080	0.162	0.434	15949.310	1
R_0	2.360	0.003	0.201	2.122	2.225	2.317	2.444	2.851	3987.971	1
recovery time	1.514	0.007	0.417	1.031	1.227	1.422	1.690	2.533	4104.463	1
incubation time	3.332	0.000	0.011	3.311	3.325	3.332	3.340	3.354	17836.610	1
i0	12.342	0.017	1.613	9.162	11.254	12.347	13.429	15.491	9331.486	1
e0	7.525	0.019	1.887	3.906	6.210	7.519	8.836	11.266	10189.980	1
ϕ	7.294	0.021	1.849	4.256	5.975	7.103	8.392	11.522	8077.164	1

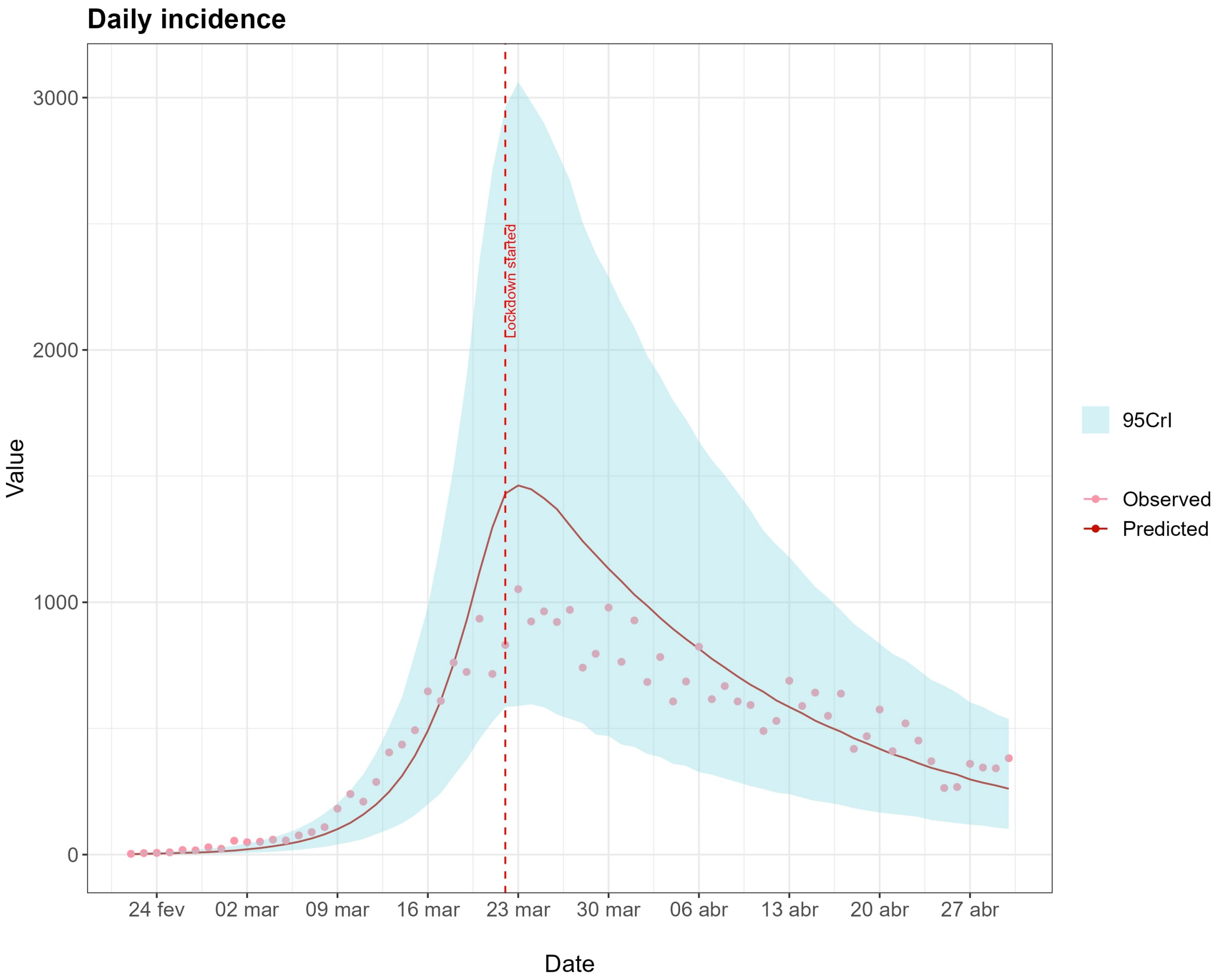

Figure 16: Observed and predicted daily absolute incidence during the study period for Scenario 6.

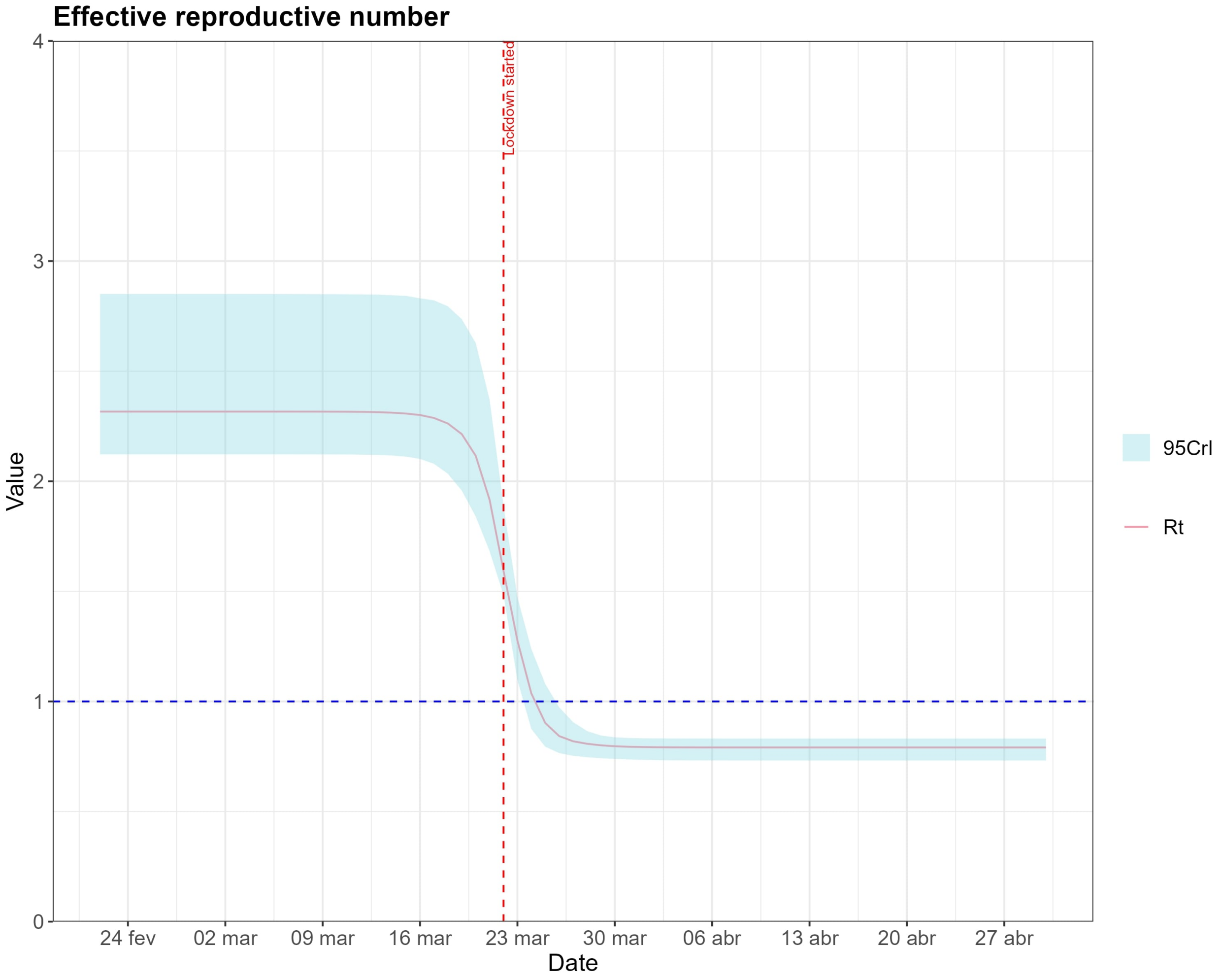

Figure 17: R_t for the Portuguese COVID-19 epidemic from 24th of February until May 1st for Scenario 6.

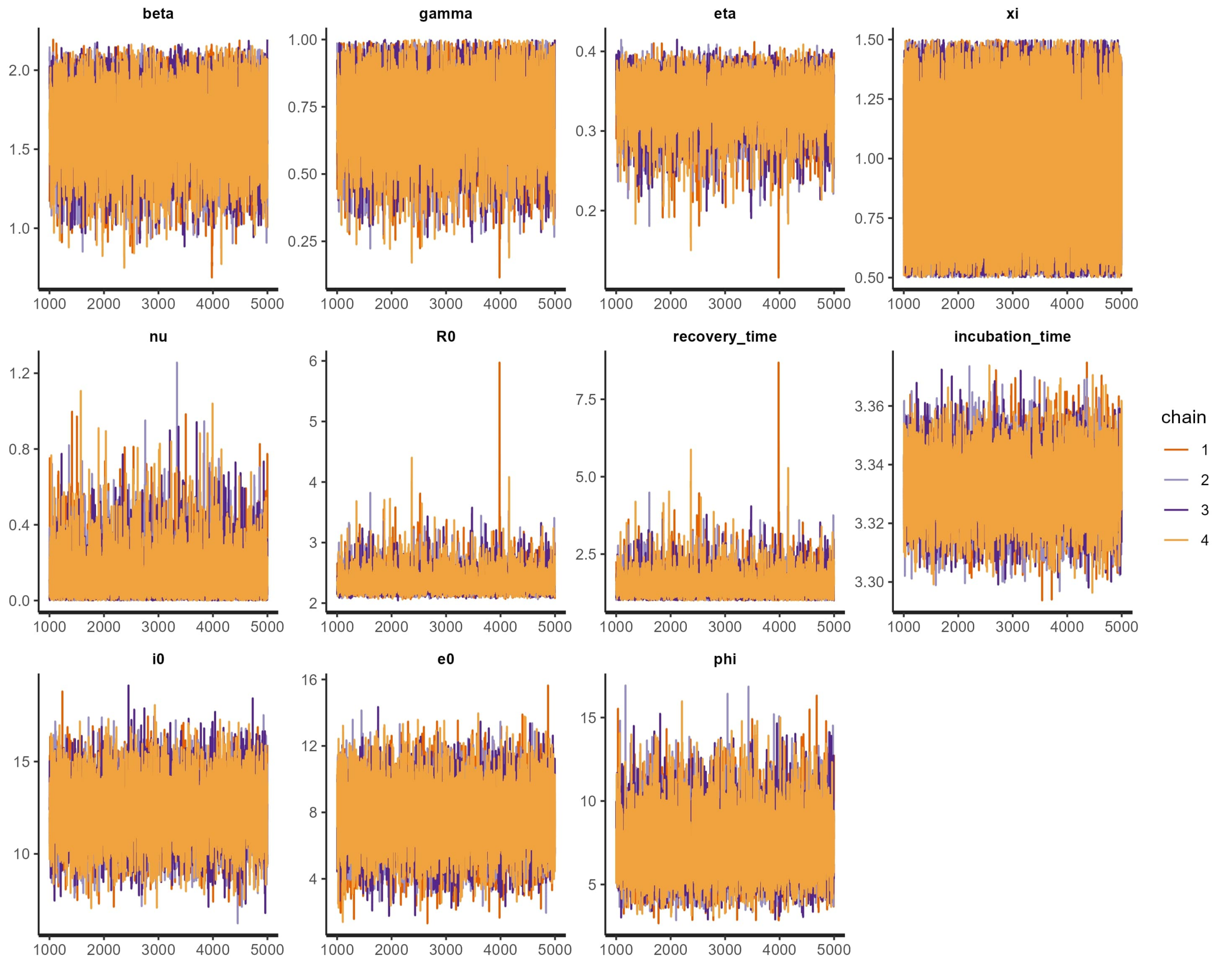

Figure 18: Posterior parameter trace plots with 4 chains and 4000 samples in Scenario 6

10 Scenario 7

Scenario 7 had a restrictive prior for i0 and e0, same with 5 and 6. The report probability was also calibrated like in Scenario 3. While this value was similar to scenario 3, and close to 36.6%, the model fit to observed data was not satisfactory.

Table 10: Posterior distributions for Scenario 7.

	mean	se_mean	sd	2.5%	25%	50%	75%	97.5%	n_eff	Rhat
β	1.62	0.003	0.252	1.124	1.444	1.624	1.808	2.068	5195.312	1
γ	0.702	0.002	0.152	0.402	0.596	0.706	0.815	0.970	5040.999	1
η	0.34	0.000	0.032	0.265	0.321	0.344	0.363	0.389	5030.091	1
ξ	0.926	0.002	0.299	0.511	0.653	0.886	1.181	1.470	15260.250	1
ν	0.116	0.001	0.118	0.003	0.033	0.081	0.162	0.430	17575.850	1
R_0	2.345	0.003	0.190	2.115	2.214	2.304	2.427	2.809	4700.258	1
recovery time	1.505	0.006	0.400	1.031	1.228	1.417	1.678	2.489	4779.872	1
incubation time	3.333	0.000	0.011	3.311	3.325	3.333	3.340	3.355	18434.570	1
i0	12.376	0.015	1.610	9.239	11.303	12.364	13.451	15.568	11061.370	1
e0	7.53	0.019	1.876	3.806	6.271	7.539	8.800	11.204	9566.379	1
ϕ	7.675	0.022	1.976	4.489	6.263	7.458	8.815	12.173	8214.046	1
%report	0.378	0.000	0.010	0.359	0.372	0.378	0.385	0.398	11840.800	1

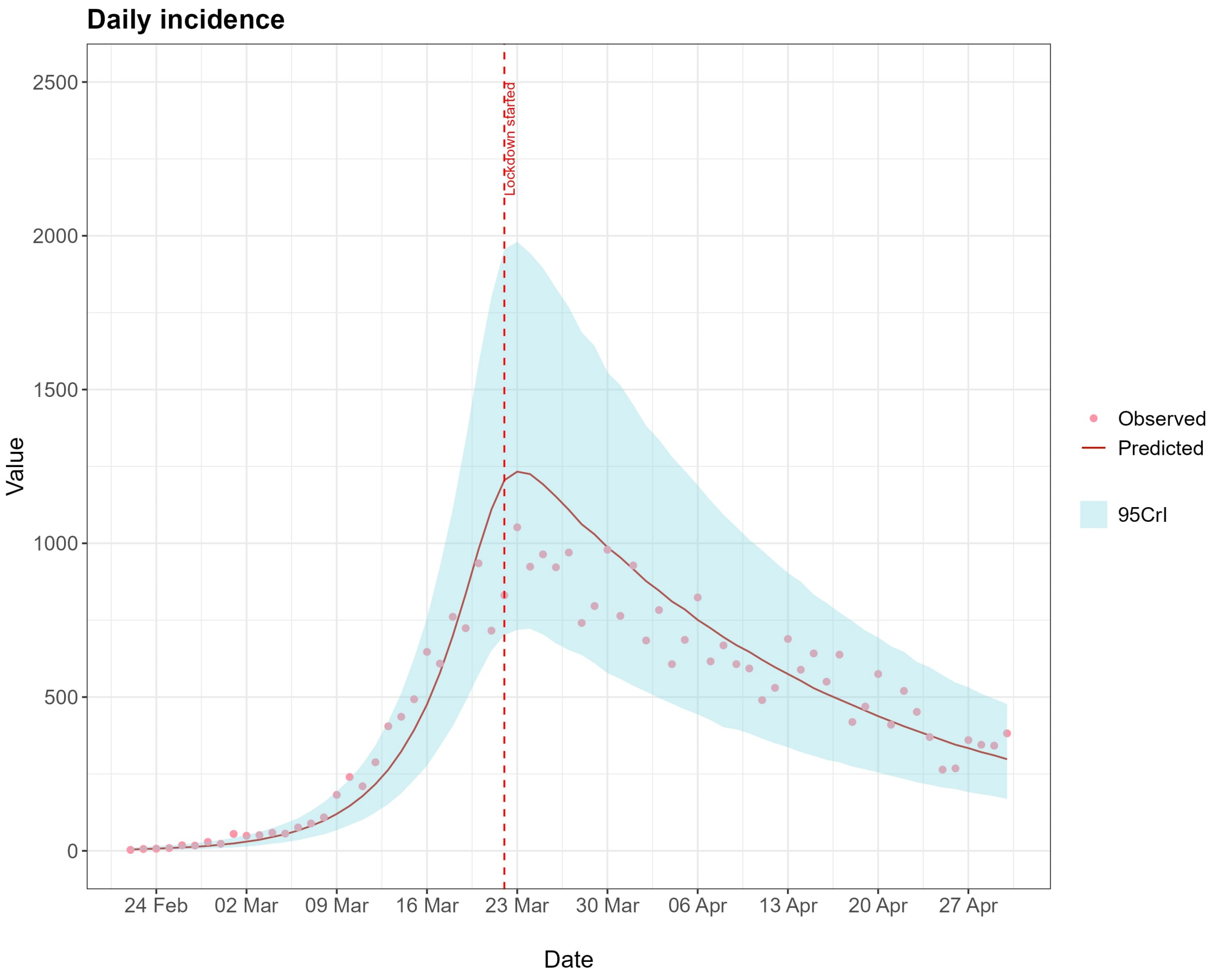

Figure 19: Observed and predicted daily absolute incidence during the study period for Scenario 7.

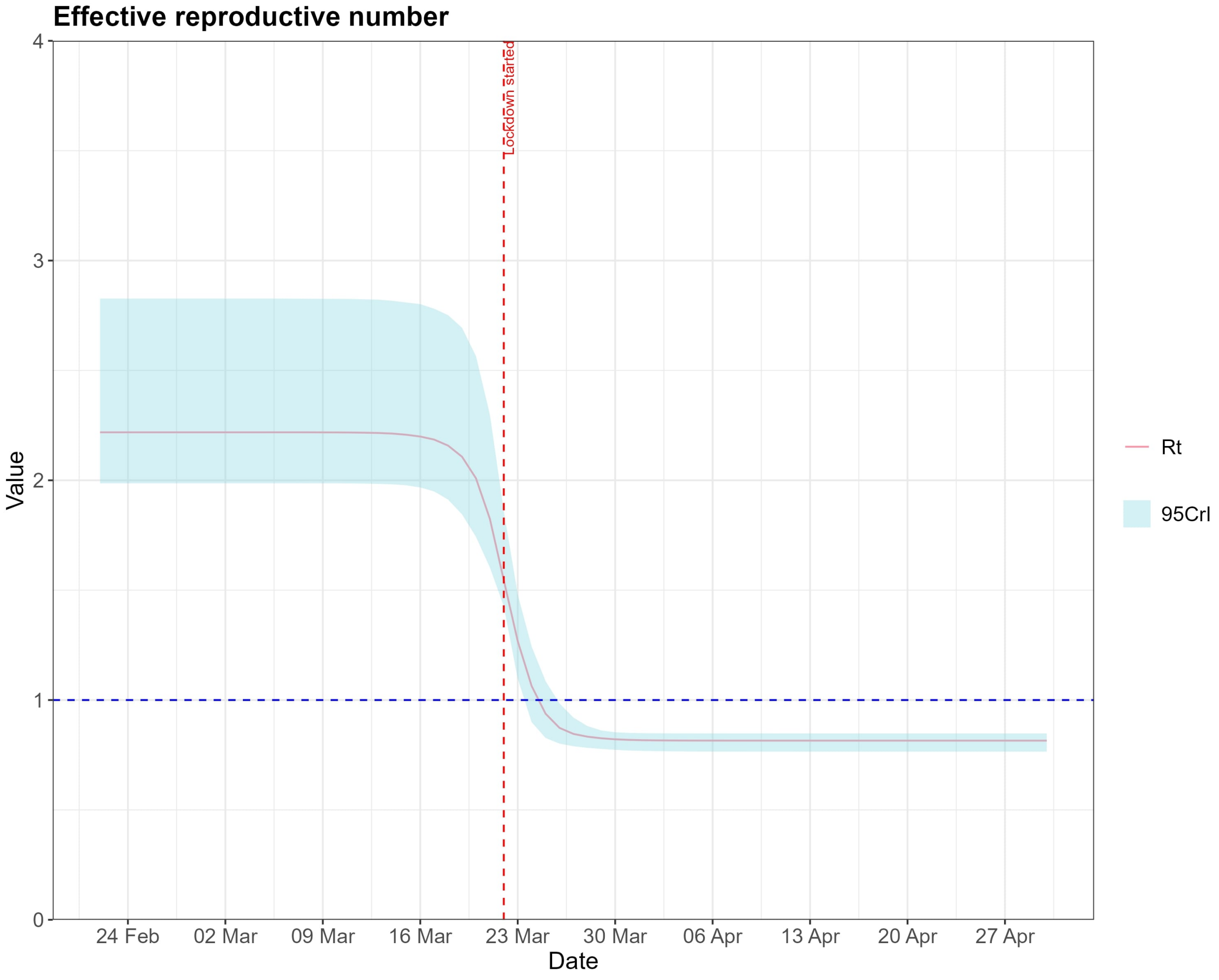

Figure 20: R_t for the Portuguese COVID-19 epidemic from 24th of February until May 1st for Scenario 7.

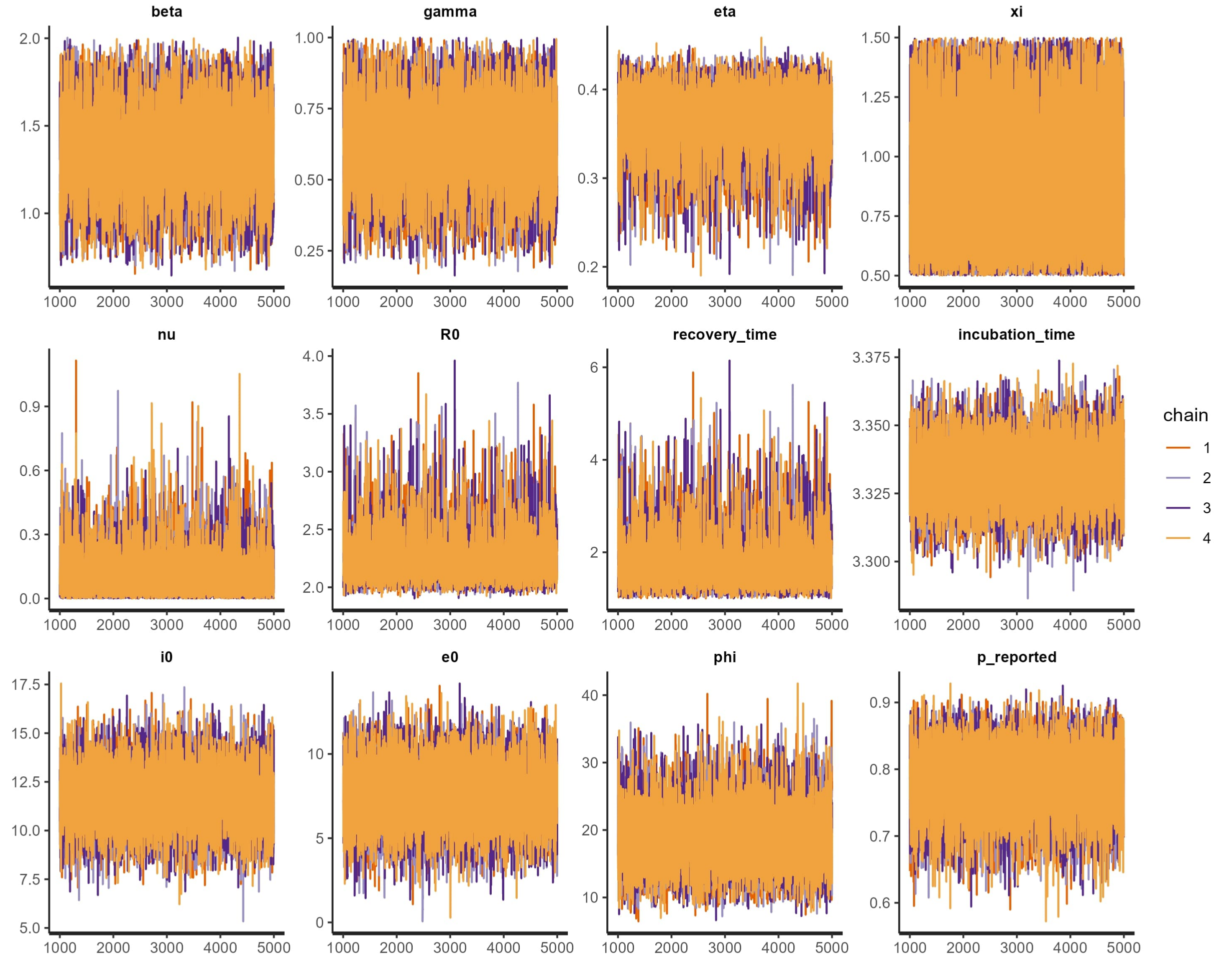

Figure 21: Posterior parameter trace plots with 4 chains and 4000 samples in Scenario 7

11 Scenario 8

In this scenario, the i0 and e0 priors was a distribution normal with mean 1 and sd 2, but the reporting probability was 100% as with Scenario 4. While the model fit to observed data was better than previous scenarios using the more restrictive prior for i0 and e0, it did not consider the underascertainment of cases.

Table 11: Posterior distributions for Scenario 8.

	mean	se_mean	sd	2.5%	25%	50%	75%	97.5%	n_eff	Rhat
β	1.167	0.004	0.247	0.751	0.985	1.146	1.326	1.704	4652.155	1
γ	0.518	0.002	0.157	0.249	0.401	0.505	0.620	0.858	4676.038	1
η	0.354	0.001	0.044	0.253	0.326	0.359	0.386	0.425	4894.077	1
ξ	0.861	0.003	0.281	0.510	0.619	0.788	1.066	1.451	11483.300	1
ν	0.095	0.001	0.094	0.002	0.027	0.067	0.133	0.343	15195.920	1
R_0	2.328	0.004	0.271	1.980	2.137	2.270	2.454	3.022	4908.157	1
recovery time	2.138	0.011	0.741	1.165	1.614	1.980	2.491	4.021	4805.212	1
incubation time	3.333	0.000	0.011	3.311	3.325	3.332	3.340	3.354	13945.070	1
i0	10.923	0.014	1.464	8.112	9.940	10.898	11.894	13.842	10597.870	1
e0	6.939	0.018	1.796	3.432	5.723	6.952	8.154	10.425	10103.630	1
ϕ	22.494	0.048	5.240	13.713	18.761	21.990	25.713	34.145	12140.980	1

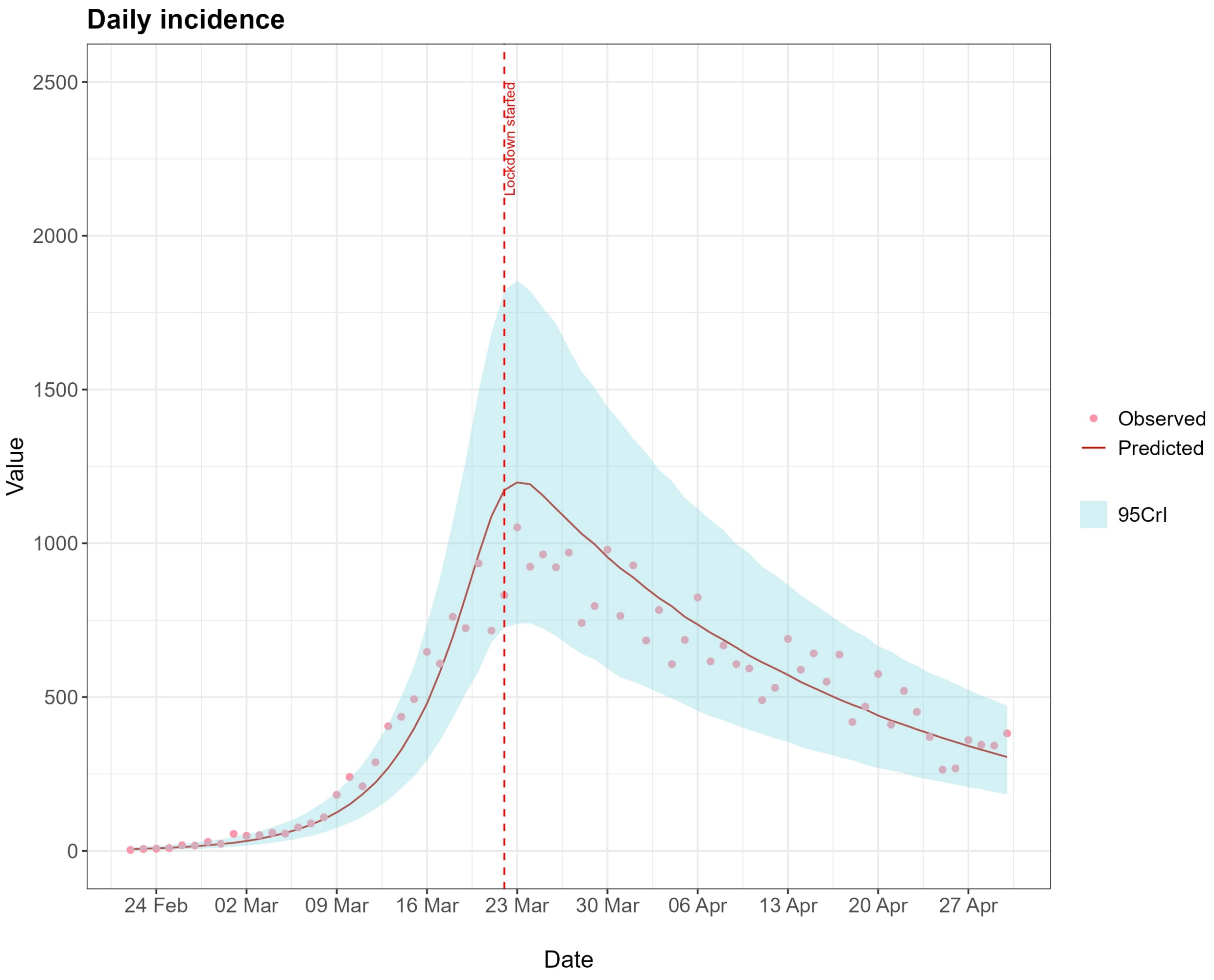

Figure 22: Observed and predicted daily absolute incidence during the study period for Scenario 8.

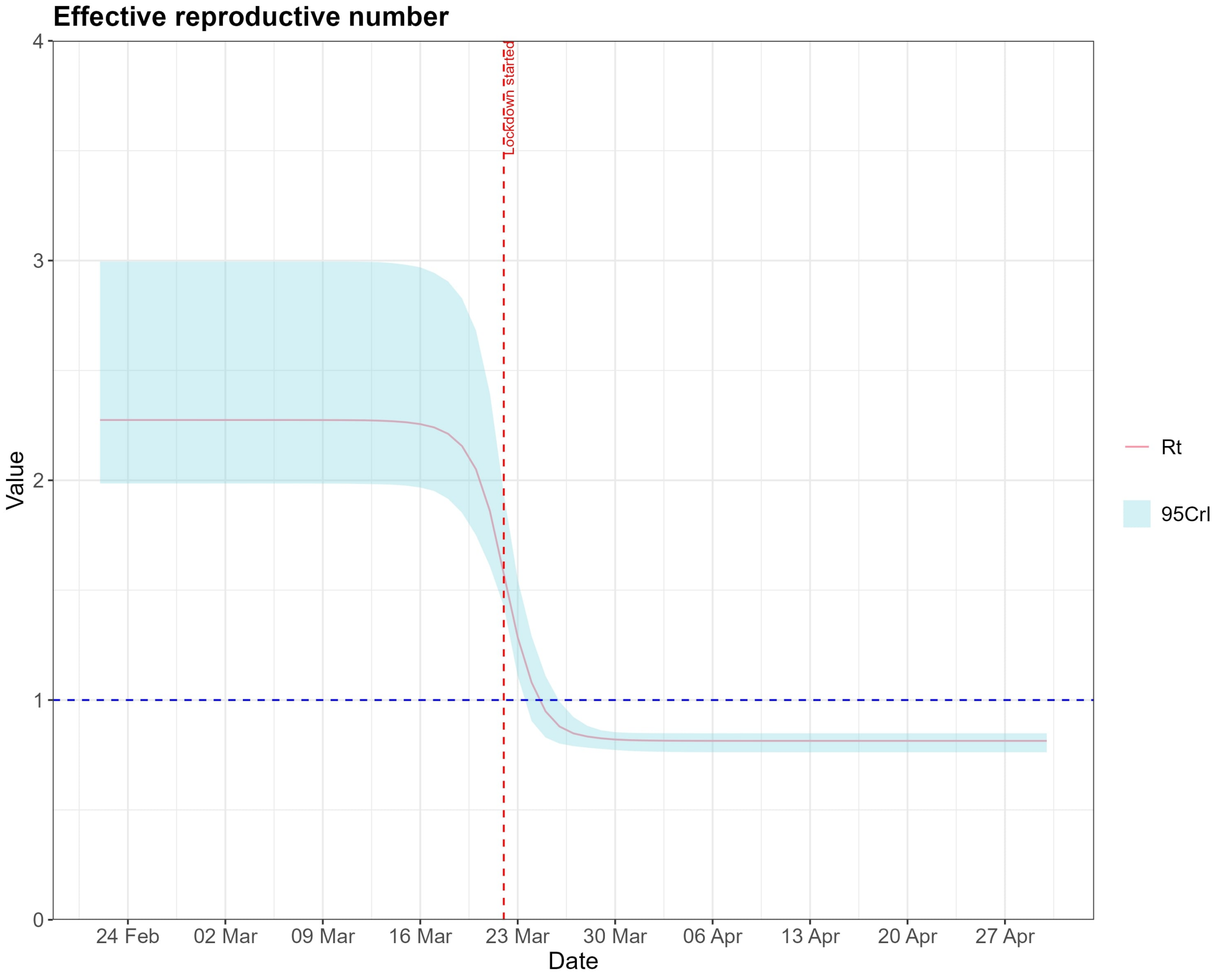

Figure 23: R_t for the Portuguese COVID-19 epidemic from 24th of February until May 1st for Scenario 8.

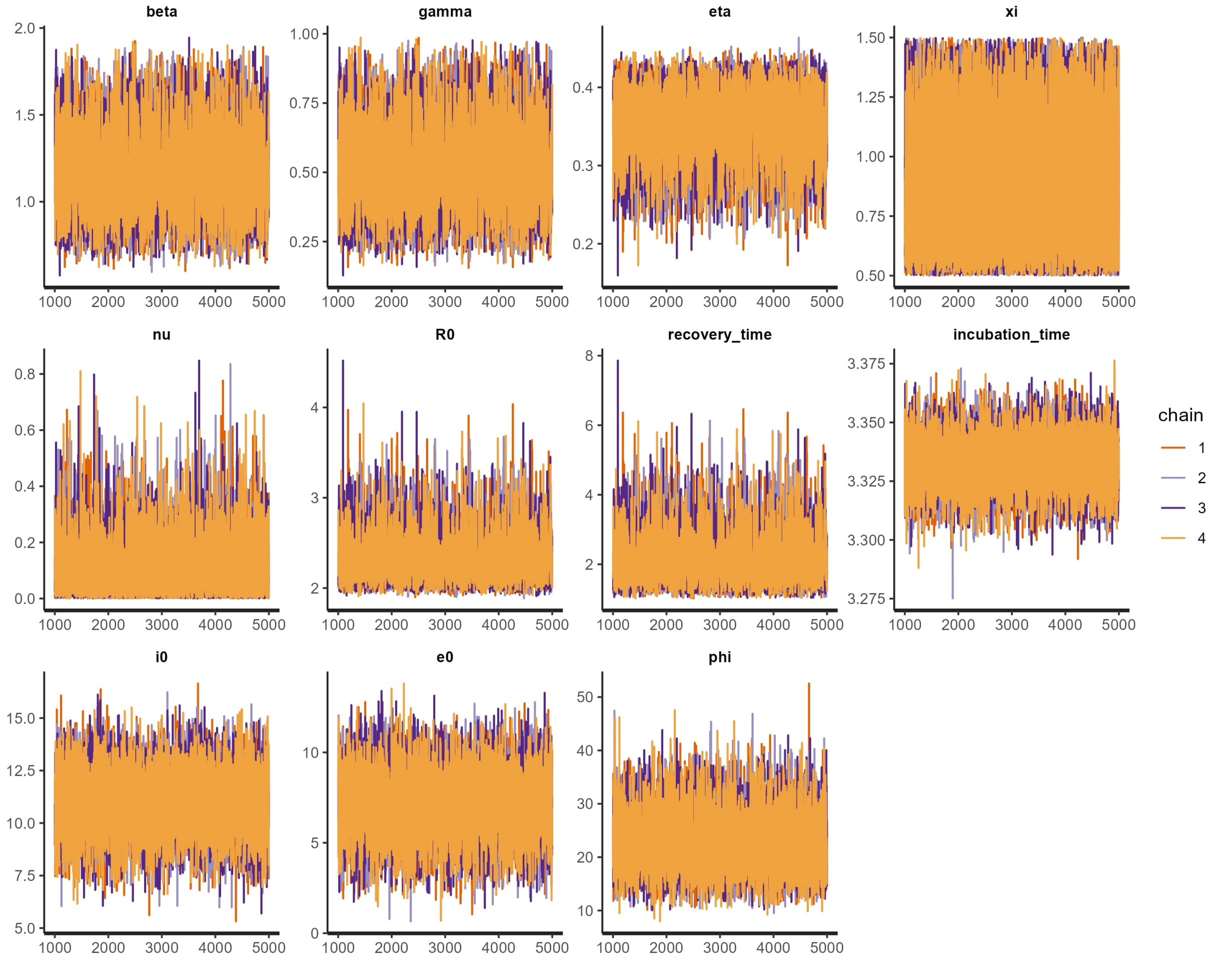

Figure 24: Posterior parameter trace plots with 4 chains and 4000 samples in Scenario 8

References

[1] Léo Grinsztajn et al. "Bayesian workflow for disease transmission modeling in Stan". In: *Stat Med* 40.27 (Nov. 30, 2021). Place: England, pp. 6209–6234. ISSN: 1097-0258. DOI: 10.1002/sim.9164.

[2] Seth Flaxman et al. "Estimating the effects of non-pharmaceutical interventions on COVID-19 in Europe". In: *Nature* 584.7820 (Aug. 2020), pp. 257–261. DOI: 10.1038/s41586-020-2405-7. URL: https://doi.org/10.1038/s41586-020-2405-7.

[3] Conselho de Ministros da República Portuguesa. *Comunicado do Conselho de Ministros de 19 de março de 2020*. 2020. URL: https://www.portugal.gov.pt/pt/gc22/governo/comunicado-de-conselho-de-ministros?i=334.

[4] Direção Geral da Saúde Portugal. Ministério da Saúde. *Norma 019/2020 "COVID-19: Estratégia Nacional de Testes para SARS-CoV-2"*. Oct. 2020. URL: https://www.dgs.pt/normas-orientacoes-e-informacoes/normas-e-circulares-normativas/norma-n-0192020-de-26102020-pdf.aspx (visited on 05/28/2024).

[5] Vasco Ricoca Peixoto et al. “Determinants for hospitalisations, intensive care unit admission and death among 20,293 reported COVID-19 cases in Portugal, March to April 2020”. In: *Eurosurveillance* 26.33 (2021), p. 2001059. DOI: 10.2807/1560-7917.ES.2021.26.33.2001059. URL: https://www.eurosurveillance.org/content/10.2807/1560-7917.ES.2021.26.33.2001059.

[6] Andrew William Byrne et al. “Inferred duration of infectious period of SARS-CoV-2: rapid scoping review and analysis of available evidence for asymptomatic and symptomatic COVID-19 cases”. In: *BMJ Open* 10.8 (Aug. 2020), e039856. ISSN: 2044-6055. DOI: 10.1136/bmjopen-2020-039856. URL: https://bmjopen.bmj.com/lookup/doi/10.1136/bmjopen-2020-039856.
